# A Novel Strategy to Predict Carcinogenicity of Antiparasitics Based on a Combination of DNA Lesions and Bacterial Mutagenicity Tests

**DOI:** 10.3389/fpubh.2017.00288

**Published:** 2017-11-09

**Authors:** Qianying Liu, Zhixin Lei, Feng Zhu, Awais Ihsan, Xu Wang, Zonghui Yuan

**Affiliations:** ^1^MOA Laboratory for Risk Assessment of Quality and Safety of Livestock and Poultry Products, Huazhong Agricultural University, Wuhan, China; ^2^National Reference Laboratory of Veterinary Drug Residues (HZAU) and MAO Key Laboratory for Detection of Veterinary Drug Residues, Wuhan, China; ^3^Department of Biosciences, COMSATS Institute of Information Technology, Sahiwal, Pakistan; ^4^Hubei Collaborative Innovation Center for Animal Nutrition and Feed Safety, Wuhan, China

**Keywords:** genotoxicity, carcinogenicity, antiparasitics, risk evaluation, DNA lesions

## Abstract

Genotoxicity and carcinogenicity testing of pharmaceuticals prior to commercialization is requested by regulatory agencies. The bacterial mutagenicity test was considered having the highest accuracy of carcinogenic prediction. However, some evidences suggest that it always results in false-positive responses when the bacterial mutagenicity test is used to predict carcinogenicity. Along with major changes made to the International Committee on Harmonization guidance on genotoxicity testing [S2 (R1)], the old data (especially the cytotgenetic data) may not meet current guidelines. This review provides a compendium of retrievable results of genotoxicity and animal carcinogenicity of 136 antiparasitics. Neither genotoxicity nor carcinogenicity data is available for 84 (61.8%), while 52 (38.2%) have been evaluated in at least one genotoxicity or carcinogenicity study, and only 20 (14.7%) in both genotoxicity and carcinogenicity studies. Among 33 antiparasitics with at least one old result in *in vitro* genotoxicity, 15 (45.5%) are in agreement with the current ICH S2 (R1) guidance for data acceptance. Compared with other genotoxicity assays, the DNA lesions can significantly increase the accuracy of prediction of carcinogenicity. Together, a combination of DNA lesion and bacterial tests is a more accurate way to predict carcinogenicity.

## Introduction

Antiparasitics are used widely throughout the world in humans and animals to kill or eliminate parasites *in vivo* and *in vitro*, and in public health to control diseases and prevent the spread of parasitism from livestock to humans. According to the pharmacological effects and the target parasite species, antiparasitics can be divided into three main groups: anthelmintics, antiprotozoal agents, and insecticides. Chemically based treatment remains the most frequently chosen tool to control parasitism. Unfortunately, the use of antiparasitics does not always result in the expected therapeutic success. The toxic effects were found to be responsible for the therapeutic failure of drug treatment (1). In the 1970s of the last century, it was reported that the chemicals had the capacity to cause cancer in both animals and humans (2, 3). Genetic and carcinogenic damage was found to have important health implications for the induction of diseases, such as lung cancer (4), pancreatic cancer (5), bladder cancer (6), leukemia (7–9), and non-Hodgkin’s lymphoma (10). Therefore, the regulatory agencies of Europe, the USA and Japan suggested that genotoxicity and carcinogenicity studies should be conducted to learn the benefit/risk ratio before commercial approval of pharmaceuticals.

It was recommended by the regulatory agencies that genotoxicity testing, which was considered to be a fundamental part of the carcinogenic risk assessment, should be performed prior to commercialization. It was forbidden to use compounds with proven genotoxic properties on humans except in rare cases with adequate justifications (11). According to the present guidelines for genotoxicity testing of pharmaceuticals (12–15), a standard test battery contains: (a) a test for gene mutations in bacteria, (b) an *in vitro* test with cytogenetic evaluation of chromosomal damage using mammalian cells or an *in vitro* mouse lymphoma thymidine kinase^±^ gene mutation assay, and (c) an *in vivo* test for chromosomal damage using mammalian hematopoietic cells. These assays were considered the best approach for genotoxic hazard identification and potential carcinogenic risk prediction. However, some limitations of this standard test battery in detecting genotoxicity were found. The current revised guidelines of the Veterinary International Conference on Harmonization and ICH S2 (R1) suggested that it can detect the genetic toxicity of most substances. However, for some special chemicals such as antimicrobial, it was required to supply the bacterial assay with a validated *in vitro* test for gene mutation in mammalian cells to detect the genetic toxicity (12, 15).

How can we identify and analyze positive genotoxicity results, especially *in vitro* cytogenetics? Two main factors including cytotoxicity and the highest testing concentration of the tested chemicals have very important effects on the result of genotoxicity. The Organization for Economic Cooperation and Development (OECD) had changed over the years to find the most suitable toxicity required at the highest concentration. In the 1999 revision, it was recommended that at least 50% toxicity should be induced. The ICH S2B suggested that *in vitro* genotoxicity tests should be conducted up to a top concentration of 10 mM in 1997 (16). In fact, when the dose level exceeds 100 µM, the physiological biological reactions will be disorder and then result in positive findings in *in vitro* genotoxicity tests. Moreover, a study sponsored by the European Center for the Validation of Alternative Methods indicated that the high testing dose should be reduced because the false-positive results in *in vitro* genotoxicity occurred at concentration levels from 1 to 10 mM. Recently, the ICH updated the genotoxicity guidelines (Table 1) (11, 17). It reduced the highest dose to 1 mM and supported the *in vivo* genotoxicity assays.

**Table 1:** Summary of the ICH (S2B) and ICH S2 (R1) proposed revision to S2.

ICH (S2B)	ICH S2 (R1)	

Bacterial mutation (Ames) (positive)	Bacterial mutation (Ames) (negative)	
	Option 1	Option 2
*In vitro* mammalian cell test (10 mM)	*In vitro* mammalian cell test [1 mM]	No requirement
Chromosome aberrations or TK gene mutation test	Chromosome aberrations or TK gene mutation test or micronucleus test	
*In vivo* cytogenetic assay	*In vivo* cytogenetic assay	*In vivo* cytogenetic assay

	Suggest to be integrated into acute toxicity assays of 28 days	

*ICH, International Committee on Harmonization of Requirements for Registration Pharmaceuticals for Human Use. It is a summary of the difference between the current ICH (S2B) guideline for testing of pharmaceuticals and the revised guideline of ICH S2 (R1) (15, 18)*.

Antiparasitics were used in the market for many years, and for a large proportion of them, genotoxicity and carcinotoxicity assays were performed prior to 1980, when the bioassays were not concordant with the present guidelines. Thus, it is necessary to re-evaluate the old data (especially the cytogenetic data) under the current guidelines of ICH S2 (R1) (17).

For pharmaceuticals, whose clinical use is continuous for at least 6 months or intermittent in chronic recurrent conditions, the long-term carcinogenicity studies in rats and mice using lifetime treatment are required (19). This has remained the most frequently chosen testing strategy since proposed by regulatory authorities in 1970s. The objective of carcinogenicity studies is to discover whether a drug has the ability to cause carcinogenicity in animals and whether this tumorigenic potential poses a relevant risk to humans (19, 20). To make an evaluation of carcinogenic risks to humans, the International Agency for Research on Cancer (IARC) in the 1–101 volumes of IARC monographs was published in the years from 1972 to 2011 (21). It examined 940 drugs in various groups: the carcinogenicity studies were sufficient for 107 drugs (11.4%), limited for 59 drugs (6.3%), and inadequate for 266 drugs (28.3%); and the remaining 508 drugs (54.0%) were not classifiable in terms of their carcinogenicity to humans. However, it included only 10 antiparasitics: 2 antiparasitics (Metronidazole and Dichlorvos) were classified as possibly carcinogenic to humans (Group 2B), and 8 antiparasitics (Chloroquine, Chlordimeform, Danex, Deltamethrin, Fenvalerate, Malathion, Permethrin, and Pyrimethamine) were considered non-classifiable in terms of their carcinogenicity to humans (Group 3).

Based on the above mentioned, it is meaningful to verify the extent of antiparasitics having the available results of genotoxicity and carcinogenicity studies. It is also necessary to re-evaluate *in vitro* genotoxicity results according to the present revised guidance. Due to the bacterial mutagenicity test alone produced misleading positive in predicting the carcinogens, we compared the combinations of bacterial mutagenicity test and other genotoxicity assays (such as cytogeneticity *in vivo* and *in vitro*, DNA lesions and mouse bone marrow micronucleus), aiming to work out a novel strategy to predict carcinogenicity.

The 136 antiparasitics that are listed in both the human andveterinary pharmacopeia were authorized by China. Forty-three and 107 antiparasitics were obtained from the human pharmacopeia and veterinary pharmacopeia, respectively. Since some parasites, including helminths, schistosome, and tapeworm, can infect both humans and animals, simultaneously, 14 antiparasitics (Albendazole, Amoscanate, Artesunate, Bithionol, Diethylcarbamazine, Ivermectin, Levamisole, Piperazine, Pyramine, Praziquantel, Mebendazole, Metronidazole, Niclosamide, and Semduramicin Soditium) can be used on both humans and animals.

The methodology of the major carcinogenicity and genotoxicity tests were summarized in Table 2. The collected information of genotoxicity and/or carcinogenicity of antiparasitics was obtained primarily from peer-reviewed journals (e.g., *Medline, Toxline*, and the *Registry of Toxic Effects of Chemical Substances*) (22), the US National Toxicology Program, the edition of *Physician*’*s Desk Reference* (23–25), the Center for Drug Evaluation and Research of the Food and Drug Administration and some relevant websites, such as http://www.updata.usa.com, http://www.osha.gov, http://www.toxnet.nlm.nih.gov, http://www.ntp.server.niehs.nih.gov, http://www.potency.berkeley.edu, http://www.fda.gov/cder, http://www.scirus.com, and http://www.inchem.org. For some antiparasitics, the genotoxicity and carcinogenicity data are incomplete in terms of the absence of the dose, the indication of an exogenous metabolic system in the genotoxicity assays, and the sex in carcinogenicity assays. In such cases, we presented our data in tables as obtained in these experimental conditions except for special markings. Moreover, regarding the present guidelines, the equivocal results that we found in extensive research were marked as positive in this review.

**Table 2:** The methodology of the major carcinogenicity and genotoxicity tests.

Test system	Materials	Principle of reference
Bacterial mutagenicity	The following fi-M Salmonella strains were used for the bacterial reverse mutation assay: TA97a, TA98, TA100, TA102, and TA1535. All strains were checked for maintenance of genetic markers prior to study	This test was performed by a plate incorporation procedure as outlined by OECD No.471, 46 Redbook 2000 IV.C.1.a (26), Redbook 2000: IV.C.1.a (27), and Chinese standard guidelines (28)
Mouse lymphoma assay	The mouse lymphoma assay using the thymidine kinase (Tk) gene of L5178Y Tk^±^ −3.7.2C mouse lymphoma cell lines was found to be the closest to the *in vivo* environment among the different *in vitro* mammalian and bacterial gene-mutation testings	The MLA was performed according to FDA toxicological principles for the safety assessment of food ingredients and OECD guidelines for the testing of chemicals. IV.C.1.c Mouse Lymphoma Thymidine Kinase Gene Mutation Assay (29) and Test Guideline 490: *In Vitro* Mammalian Cell Gene Mutation Tests Using the Thymidine Kinase Gene (30)
Chromosomal aberration assay	The potential of tested compound to induce structural and numerical chromosome aberrations was evaluated in Chinese hamster lung fibroblast cells (V79)	Chromosomal aberration assay *in vitro* according to OECD No.473 (31), Redbook 2000 IV.C.1.b *In Vitro* Mammalian Chromosomal Aberration Test (32)
Bone marrow erythrocyte micronucleus assay	For each treated animal, at least 1,000 polychromatic erythrocytes (PCE) were counted to determine the micronucleus frequencies and record the micronucleus occurrence rate per one thousand PCE, and the proportion of PCE to normochromatic erythrocytes (NCE) was evaluated by counting a total of 1,000 erythrocytes	This assay was conducted in accordance with OECD Guideline No.474 (33) and Redbook 2000 IV.C.1.d. Mammalian Erythrocyte Micronucleus Test (27)
HGPRT mutation test	Mutations were expressed during a period of 6–7 days, including two subculturing steps. Subsequently, mutant frequencies (mutants/106 cells) and cloning efficiencies were scored	This assay was carried out following standard test procedures (34)
Unscheduled DNA synthesis assay	Prior to drug treatments, peripheral blood lymphocytes were isolated from healthy individuals. The radioactivity was determined by Beckman Ls3801 liquid scintillation spectrometry	This assay was performed according to the OECD guideline number 482 (26, 34)
Long-term carcinogenesis assay in rodent	The animal were randomly assigned to four groups based on their body weights, and each group of animal were fed the basal diet mixed with tested compound for a total period of 78 weeks (mice) and 104 weeks (rat)	Long-term carcinogenesis assay was conducted according to the guidelines of Ref. (35, 36)

## Results

### Genotoxicity and Carcinogenicity of Antiparatics

For the present analyses, an antiparasitic was regarded as genotoxic when it produced positive or equivocal results in at least one of the standard battery tests, and as a rodent carcinogen when it increased tumor incidence. Table 3 covers the information available on genotoxicity and carcinogenicity findings for each tested antiparasitic. The following genotoxicity assays were used: Ames (bacterial mutagenesis), sex-linked recessive lethal, *in vitro* cytogenetics (chromosome aberrations), *in vivo* cytogenetics [chromosome aberrations, micronucleus and sister chromatid exchange (SCE)], unscheduled DNA synthesis *in vitro* (UDS), MLA (mouselymphoma L5178Y TK^±^ assay), and other types of genotoxicity studies, including DNA fragmentation, mammalian mutagenesis HGPRT, SCE *in vitro*, DNA strand break analysis *in vitro*, and the micronucleus assay *in vitro*. The long-term carcinogenicity test was carried out in mice, rats, and other species.

**Table 3:** Genotoxic and carcinogenicity effects of antiparasitics.

Test system	Dose or concentration (LED or HID)	Result	Reference
**1. Acriflavine (8048-52-0)**			
*Salmonella typhimurium* (none), TA1537, TA1538, TA98	50 μg/plate	−	(37)
*Salmonella typhimurium* (rat, liver, S-9, aroclor1254), TA1537, TA1538, TA98	50 μg/plate	+	(37)
Gene mutation, *Aspergillus nidulans*		+	(38)
Chinese hamster ovary (CHO), CHO-K1-BH4 (HGPRT)	0.5–4 µg/l	+	(39)
Chromosome aberrations *in vivo*, Mammalian or early embryo		−	(40)
Forward and reverse gene mutation, host-mediated assay, *Salmonella typhimurium*#		−	(41)
Sex-linked recessive lethals and sex-chromosome loss		+	(42)
Micronucleus test *in vivo and in vitro*, chromosome aberrations, mammalian polychromatic erythrocytes, mammalian cell culture, non-human		+	(43)
Mitotic recombination or gene conversion, *Saccharomyces cerevisiae*		NC	(44)
Sperm morphology, mouse		+	(45, 46)

**2. Albendazole (54965-21-8)**			
Bacterial mutation (Ames)		−	(47)
SCE and micronucleus (MN) on human lymphocytes *in vivo*	15 mg/kg p.o. in diet for 28 days	+	(48)
Micronuclei in cultured peripheral blood lymphocytes *in vitro* and in cultured human lymphocytes	10–100 µg/ml	+	(49)
Cytogenetics *in vitro* and *in vivo*		−	(47)
Micronucleus assay with CHO-K1 cells *in vitro*		+	(50)
Long-term carcinogenesis assay, mice	400 mg/kg/day	−	(24)
Long-term carcinogenesis assay, rats	20 mg/kg/day	−	(24)

**3. Amitraz (33089-61-1)**			
*Salmonella typhimurium*, TA98, TA100, TA97, TA102	0–200 μg/plate	−	(51)
Genotoxic in the vibrio test	10^−3^ to 10^−5^ µg/plate	−	(52)
DNA damage on hamster cells *in vitro*, comet assay	3.75 µg/l	+	(53)
Long-term carcinogenesis assay. rat (oral)	0, 15, 50, 200 mg/l in feed for 104 weeks	−	(54)
Long-term carcinogenesis assay. mouse (oral)		−	(54)

**4. Amodiaquine (86-42-0)**			
*Salmonella typhimurium*, TA100, reverse mutation	0.1–5,000 μg/plate	−	(55)
*Salmonella typhimurium*, TA97A, TA102, TA104	0.1–1,000 μg/plate	−	(56)
*Salmonella typhimurium*, TA100 (rat, liver S-9, Phenobarbital), reverse mutation	0.1–5,000 μg/plate	−	(56)
*Salmonella typhimurium*, TA97A, TA102, TA104 (rat, liver S-9, Phenobarbital), reverse mutation	0.1–1,000 μg/plate	−	(56)

**5. Amoscanate (26328-53-0)**			
*Salmonella typhimurium*, TA1537, TA1535, TA100, TA1538, TA98, reverse mutation	0.1–1,000 μg/plate	−	
*Salmonella typhimurium*, TA1537, TA1535, TA100, TA1538, TA98 (rat, liver S-9, aroclor 1254 or Phenobarbital), reverse mutation	0.1–1,000 μg/plate	−	
*Salmonella typhimurium*, TA100 (rat, liver S-9, aroclor 1254 or Phenobarbital), reverse mutation	20–160 nmol/plate	−	(57)

**6. Amphotericin B (1397-89-3)**			
Bacterial mutation (Ames)		−	(47)
Chromosome aberrations, peripheral blood lymphocytes		−	(58)
Cytogenetics *in vitro* and *in vivo*		−	(47)
MLA		−	(47)

**7. Atovaquone (95233-18-4)**			
Bacterial mutation (Ames)		−	(47)
Cytogenetics *in vitro* and *in vivo*		−	(47)
MLA		−	(47)
Long-term carcinogenesis assay, mice (liver tumors)	human AUC × 5	+	(24)
Long-term carcinogenesis assay, rats	NR	−	(24)

**8. Bithionol (97-18-7)**			
*Salmonella typhimurium* (none), TA98, TA100, TA97, TA102, TA100, TA1535, TA1537, TA97	0.1–1,000 μg/plate	−	(59)
	0.1–6.6 µg/plate	−	(60)
*Salmonella typhimurium* (rat, liver, S-9, kanechlor 400) TA98, TA100, TA97, TA102	0.1–1,000 μg/plate	−	(59)
*Salmonella typhimurium* (Hamster, liver, S-9, Aroclor 1254) TA100, TA1535, TA97, TA98	1–200 μg/plate	−	(60)
Micronucleus test *in vivo*, chromosome aberrations, mammalian polychromatic erythrocytes		−	(61)

**9. Bromofenofos (21466-07-9)**			
*Salmonella typhimurium* (rat, liver, S-9, kanechlor 400), TA100, TA98, TA1535, TA1537, TA1538; *Salmonella typhimurium* (none), TA100, TA98, TA1535, TA1537, TA1538	0.005–0.5 mg/plate	−	(61)
Micronucleus test *in vivo*, chromosome aberrations, mammalian polychromatic erythrocytes (mouse)		−	(61)

**10. Chlordimeform (6164-98-3)**			
*Salmonella typhimurium*, TA1535, TA1537, TA98, TA100	1–7,500 μg/plate	−	(62)
*Salmonella typhimurium*, TA98, TA100, TA1535, TA1537, TA1538	1–2,000 μg/plate	−	(63)
			(64)
Recombination assay, *Bacillus subtilis* (H17 vs. M45)		−	(65)
			(63)
*E. coli* polA (WP_2_ uvra), recombination assay, DNA effects (bacterial DNA repair)	10^−5^ g/ml	−	(65)
*E. coli*	1–7,500 μg/plate	−	(62)
UDS *in vitro*, DNA effects (Human diploid fibroblasts FL cell)	10^−6^ to 10^−3^ g/ml	−	(66)
		+	(66)
Chromosomal aberrations *in vitro* and *in vivo* human peripheral lymphocytes	MTD	−	(67)
Chromosomal aberrations *in vivo*, Chinese hamster cells (CHO), Voles living donor bone marrow cells	MTD	−	(68)
SCE, bone marrow cells in mice, Voles living donor bone marrow cells, Voles fibroblasts	10 mg/kg	+	(67)
	80 mg/kg	+	(63)
Micronucleus test, mice bone marrow cells *in vivo*, peripheral lymphocytes	77 mg/kg	−	(69)
Mitotic recombination or gene conversion, *Saccharomyces cerevisiae*		−	(44)
Neoplasms		+	(70)
Carcinogenicity studies in mouse and rat		+	(71)
Chromosomal aberrations, mouse bone marrow cells *in vivo*	100 mg/kg	+	(55)

**11. Chloroquine (54-05-7)**			
*Salmonella typhimurium*, TA97, TA1537, reverse mutation	250 µg/plate	+	(72, 73)
	200 µg/l		
*Salmonella typhimurium*, TA1977, TA1535, TA1537, TA1538, reverse mutation	600 µg/l	−	(74, 75)
	10 000 µg/plate		
*Salmonella typhimurium*, TA98, TA100, reverse mutation	0–10,000 µg/plate	+	(56, 73)
*Salmonella typhimurium*, TA98, TA100, TA1537, TA1538, reverse mutation	5,000 µg/plate	NC	(73, 76)
*Salmonella typhimurium*, TA97A, TA1537, reverse mutation	5,000 µg/plate	−	(73, 77)
*Salmonella typhimurium*, TA98, TA100, TA97A, TA100, reverse mutation	50 µg/plate	−	(73, 78)
	10,000 µg/plate		
*Salmonella typhimurium*, TA102, TA104, reverse mutation	5,000 µg/plate	−	(56, 73)
*E. coli* WP2 uvra, reverse mutation	5,000 µg/plate	NT	(72, 73)
*E. coli*, reverse mutation	300 µg/plate	+	(78)
*Salmonella typhimurium*, TA97A, TA100, reverse mutation	20–50 µg/plate	+	(78)
*Salmonella typhimurium*, TA97A, TA100 (rat, liver S-9, phenobarbital); *Salmonella typhimurium*, TA102, TA104; *Salmonella typhimurium*, TA102, TA104 (rat, liver S-9, phenobarbital), reverse mutation	0.1–10,000 µg/plate	−	(79)
*E. coli polA* (W3119 vs. P3478) Rec-assay, DNA effects (bacterial DNA repair)	0.1–10,000 µg/plate	+	(55)
Chromosome aberrations, mammalian cell culture, non-human, micronucleus test *in vitro*		+	(43)
SCE, mouse bone marrow cells *in vivo*	12.5 mg/kg	+	(78)
Chromosomal aberrations, mouse bone marrow cells *in vivo*	100 mg/kg	+	(55)

**12. Closantel (57808-65-8)**			
Chromosomal aberrations *in vivo*, bone marrow cells	0, 5, 10, 15, 20 mg/kg	+	(80)

**13. Coumaphos (56-72-4)**			
*Salmonella typhimurium* (none), TA98, TA1535, TA1537, TA1538, TA100, TA100, TA98	3.3–3333.3, 3.3–10,000, 0.3–333.3 µg/plate	−	(81)
*Salmonella typhimurium* (rat, liver, S-9, aroclor 1254), TA98, TA1535, TA1537, TA1538, TA100, TA100, TA98	3.3–3333.3, 3.3–10,000, 0.3–333.3 µg/plate	−	(81)
*Salmonella typhimurium* (none), TA98, TA100, TA1535, TA1537, TA1538	667, 1.000, 3.333, 6.667, 10,000 µg/plate	−	(82)
*E. coli* WP2 uvra, (none); *E. coli* WP2 uvra (rat, liver, S-9, aroclor 1254)	3.3–10,000, 0.3–333.3 µg/plate	−	(81)
*E*. *coli*, mouse, liver, S-9; *E. coli*, hamster liver, S-9, aroclor 1254	3.3–10,000, 0.3–333.3 µg/plate	−	(81)
Chromosomal aberrations *in vitro*, CHO cells (rat, liver, S-9, aroclor 1254)	100, 300, 1,000 µg/l	−	(83)
Chromosomal aberrations *in vitro*, CHO cells (none)	99.5, 299, 995 µg/l	−	(83)
Micronucleus *in vivo*, polychromatic erythrocytes	480 mg/kg of coumaphos at 98.0% purity	+	(82)
Carcinogenicity studies, rats	0 (1% peanut oil), 1, 5, 25 mg/l in diet for 24 months	−	(82)
Carcinogenicity studies, mouse	0, 10, 20 mg/l in diet	−	(84, 85)
Carcinogenicity studies, rats	0, 10, 20 mg/l in diet	−	(84, 85)

**14. Cyfluthrin (68359-37-5)**			
*Salmonella typhimurium*, TA98, TA100 (none); TA98, TA100 (rat liver S9), reverse mutation	1,000–5,000 µg/plate	−	(86)
Gene mutation, Ames/micronucleus test in cultured human peripheral blood lymphocytes		−	(86)
Chromosomal aberrations in cultured human peripheral blood lymphocytes; chromosomal aberrations *in vivo*	1,000, 2,000 mg/ml	+	(86)
	250, 500, 1,000 mg/kg b.w.		
SCE, in cultured human peripheral blood lymphocytes	500, 1,000, 2,000 mg/ml	−	(86)
SCE in blood lymphocytes	500, 1,000, 2,000 µg/l		
Micronucleus (MN) formation in cultured human peripheral blood lymphocytes	500, 1,000, 2,000 mg/ml	+	(86)
DNA damage on the epithelial cells of human nasal mucosa	0.05, 0.1, 0.5, 0.75, 1.0 mg/ml	+	(87)
DNA damage and comet assay in fish species	5.6 mg/l beta-cyfluthrin for 48 h	+	(88)
Chromosomal aberrations *in vitro*	500, 1,000, 2,000 µg/l	−	(86)
Mouse bone marrow cells *in vitro*	1,000 µg/l	+	(86)

**15. Cypermethrin (52315-07-8)**			
*Salmonella typhimurium*, TA98, TA100, TA1535		−	(89)
Micronuclei formation in bone marrow cells in rats; DNA damage in blood cells in rats	25 mg/kg b.w. p.o. for 28 days	+	(90)
Micronucleus test in mice *in vivo*		NC	(91)
Chromosomal aberrations (CAs) on human peripheral lymphocytes; SCE on human peripheral lymphocytes	12.5 + 2.5, 15 + 5, 17.5 + 7.5, 20 + 10 mg/ml	+	(92)
Micronucleus (MN) tests on human peripheral lymphocytes	12.5 + 2.5, 15 + 5, 17.5 + 7.5 mg/ml	+	(92)
Excision-repairable DNA damage in ICR mouse hepatocytes		−	(93)
DNA strand breakage and DNA hypomethylation in ICR mouse hepatocytes		+	(93)
Chromosomal aberrations on human peripheral lymphocytes	5, 10, 15, 20 mg/ml	+	(94)
SCE on human peripheral lymphocytes			
Micronucleus (MN) tests on human peripheral lymphocytes	5, 10 mg/ml	+	(94)
Chromosomal aberration (CA) in highly mitotic kidney cells; micronucleus (MN) tests in erythrocytes of a freshwater fish	0.4, 0.8, 1.2 µg/l for 48 and 72 h	+	(95)
DNA damage in vital organs in mouse	12.5, 25, 50, 100, 200 mg/kg b.w.	+	(96)
DNA damage using alkaline comet assay	25, 50, 75 mg/kg b.w. for 6–15 days	+	(97)
Transplacentally genotoxic			
Peripheral blood for MN test	20, 30, 40, 50 mg/l	+	(98)
Excision repairable DNA lesions		−	(99)
Long-term carcinogenesis assay, rat	75, 1,500 mg/kg b.w.	−	(100)
Long-term carcinogenesis assay, mouse	240, 1,600 mg/kg b.w.	−	(100)

**16. Danex (52-68-6)**			
*E. coli*, WP2 (rat, liver S-9, aroclor 1254)	500–10,000 µg/plate	+	(101)
*E. coli*, WP2 UVRA (rat, liver S-9, aroclor 1254)			
UDS Human fibroblasis			(66)
*Salmonella typhimurium*, TA100, reverse mutation	1–5,000 µg/plate	+	(101)
*Salmonella typhimurium*, TA1535, TA1535 (rat, liver S-9, aroclor 1254), reverse mutation	1.25–5,000 µg/ml	−	(102)
*Salmonella typhimurium*, TA104, TA100 (rat, liver S-9, aroclor 1254), reverse mutation	5–25 mg/plate	+	
*Salmonella typhimurium*, TA104, TA100, TA1535, TA97, reverse mutation	1–25 mg/plate	+	(102)
*Salmonella typhimurium*, TA1535, TA97 (rat, liver S-9, aroclor 1254), *Salmonella typhimurium*, TA100, TA98, TA104		−	(102)
*Salmonella typhimurium*, TA100, TA98, TA97; *Salmonella typhimurium*, TA100, TA98, TA104 (rat, liver S-9, aroclor 1254)	0.1–25 mg/plate	−	(102)
*Salmonella typhimurium*, TA100, TA98, TA97, TA1535, TA1537 (rat, liver S-9, aroclor 1254), reverse mutation	500–5,000 µg/plate	−	(102)
*Salmonella typhimurium*, TA1535, TA1537, reverse mutation	100–10,000 µg/plate	−	(102)
*Salmonella typhimurium*, TA98, TA100, reverse mutation	33–10,000 µg/plate	−	(103)
Chromosomal aberrations, V79	0.4–4,000 mmol	−	(104)
	0.04–0.8 mmol	+	
Micronucleus *in vivo*, mouse	100 or 200 mg/kg	+	(105)
	3.13, 6.25, 12.5, 25 mg/kg		(106)
UDS human cells		−	(103)

**17. Deltamethrin (52918-63-5)**			
*Salmonella typhimurium*, TA98, TA100, TA1535, TA1537, and TA1538		−	(70)
*Salmonella typhimurium*, TA98, TA100	20–600 µg/plate	−	(107)
*Salmonella typhimurium*, TA98, TA100, TA1535, TA1537, TA1538	0–5,000 µg/plate	−	(108)
Chromosomal aberrations, CHO cells *in vitro*	0, 19, 38, 75, 150 µg/l	+	(108)
Micronucleus test, mice bone marrow cells *in vivo*	8.0–90.0 mg/kg	+	(109)
V79/6-thioguanine, Chinese hamater V79	4–40 µg/l	−	(107)
Carcinogenesis assay. mouse (dermal)	0, 1, 2,4 mg/kg b.w. for 32 weeks	−	(110)
Long-term carcinogenesis assay. Rat (intragastric)	0, 3, 6 mg/kg for 120 weeks	−	(111)
Long-term carcinogenesis assay. Rat (oral)	0, 25, 125, 500, 800 mg/l in feed for 2 years	−	(108)
Long-term carcinogenesis assay. Mouse (oral)	0, 10, 100, 1,000, 2,000 mg/l in feed for 97 weeks	−	(108)
Long-term carcinogenesis assay. Mouse (intragastric)	0, 1, 4, 8 mg/kg in diet for 120 weeks	−	(111)

**18. Diaveridine (5355-16-8)**			
Bacterial umu test, *S. typhimurium*, TA1535	0.1, 0.3, 1.0, 3.0 µg/l	−	(112)
*Salmonella typhimurium*, TA100, TA98, TA97, TA102	0.5, 1.0, 2.5, 5.0, 10, 25 µg/plate	−	(112)
*E. coli*, WP2 uvra/pkm101	0.5, 1.0, 2.5, 5.0, 10, 25 µg/plate	−	(112)
Chromosome aberration in cultured Chinese hamster CHL cells	12.5, 25, 50, 100 µg/l	+	(112)
Micronucleus test in rodent bone marrow, mice and rats	500, 1,000, 1,500, 2,000 mg/kg b.w.	−	(112)
Comet assay in five mouse organs *in vivo*	1,000, 1,500, 2,000 mg/kg b.w.	+	(112)
*Salmonella typhimurium*, TA98 (rat, liver, S9)		−	(113)
*Salmonella typhimurium*, TA98 (Hamster, liver, S9), TA100 (rat, liver, S9) reverse mutation		−	(113)
*Salmonella typhimurium*, TA100 (Hamster, liver, S9)		+	(113)
*Salmonella typhimurium*, TA97, TA98, TA100, TA102 (rat, liver, S9) reverse mutation	0.1–3.0 µg/l	−	(112)
*Salmonella typhimurium*, TA1535, TA1535 (rat, liver, S9)	10 µg/l	−	(112)
Chromosomal aberrations	100 µg/l,48 h	+	(112)
Mouse bone marrow cells *in vivo*, rat		−	(112)
Comet assay (liver, kidney, lung, spleen)		+	(112)
Comet assay (bone marrow)		−	(112)

**19. Diazinon (333-41-5)**			
*Salmonella typhimurium*, TA1535, TA1536, TA1537, TA1538 carcinogenicity studies *in vivo*			(114)
*Salmonella typhimurium* (TA98, TA100, TA1535, TA1537, and TA1538), reverse mutation		−	(115, 116)
*Salmonella typhimurium*, TA98, TA97, TA102, TA1535, TA1537, TA100 reverse mutation	20–80 mg/l, 100–10,000 µg/plate	−	(117, 60)
*E. coli* WP2 uvra, tryptophan reverse gene mutation		−	(101)
*E. coli* (rat, liver S-9, aroclor 1254), mouse, Hamster	0.3–333.3, 1–100, 10–10,000 µg/plate	−	(81)
MNs (micronuclei) in rat lymphocytes	150 mg/kg b.w.	+	(118)
SCE, non-human CHO cells *in vitro*		+	(119)
SCE, human Laz-007 B lymphoid cells *in vitro*		+	(120)
DNA effects (bacterial DNA repair), *Bacillus subtilis* (H17 vs. M45), recombination assay,		NC	(79)
DNA damage in human blood lymphocytes *in vitro*	750 µg/l	+	(121)
UDS *in vitro*, DNA effects human diploid fibroblasts		−	(66)
Mitotic recombination or gene conversion, *Saccharomyces cerevisiae*		−	(44)
Long-term carcinogenesis assay. mice	0, 100, 200 mg/l in diet	−	(122, 123)
Long-term carcinogenesis assay. rats	0, 400, 800 mg/l in diet		(84, 85)

**20. Dichlorvos(DDVP) (62-73-7)**			
*Salmonella typhimurium*, TA100	500–1,000 µg/plate	+	(124)
	100–6,666 µg/plate	+	(125)
	0.5–500 µg/plate	++	(126)
	100–5,000 µg/plate	+	(60)
	100–1,000 µg/plate	+	(127)
*Salmonella typhimurium*, TA98	100–6,666 µg/plate	+	(125)
	100–5,000 µg/plate	+	(60)
*Salmonella typhimurium* (TA98, TA100, TA1535, TA1537, TA1538), histidine reverse gene mutation		+	(115, 116)
*Salmonella typhimurium*, forward and reverse gene mutation, mitotic recombination and gene conversion, DNA effects, host-mediated assay		−	(128)
SCE *in vitro*, human lymphocytes		−	(129)
SCE *in vitro*, non-human	With dose response	+	(130)
SCE *in vitro*, human, human lymphocytes		NC	
*E*. coli (rat, liver S-9, aroclor 1254)	22.6 µg/l	+	(101, 131)
*E. coli* polA (W3119 vs. P3478), Recombination assay, DNA effects (bacterial DNA repair)		+	(79)
*E. coli* WP2 uvra, tryptophan reverse gene mutation		+	(101)
*E. coli*	5 mg/ml	+	(132)
		+	(101)
		+	(133)
Chromosome aberrations, mammalian polychromatic erythrocytes		NC	(134)
Chromosomal aberrations *in vitro*, CHO cells	16, 50, 100, 160 µg/l	+	(135)
	50, 160, 500, 1,600 µg/l	+	
	500, 750, 1,000 µg/l	+	
Chromosome aberrations, *Allium cepa*	With dose response	+	(136)
Chromosome aberrations, non-human bone marrow *in vivo*		−	(40)
Chromosome aberration, mammalian germ cells *in vivo*		−	(137)
Chinese hamster V79	1.25–5 µg/l	−	(104)
CHO, CHO-k1-bh4 (HGPRT)/6-thioguanine	50–150 µg/l	+	(138)
Mitotic recombination or gene conversion, *Saccharomyces cerevisiae*		+	(44)
Mouse lymphoma, L5178Y (TK+/TK−)	0–0.33 µg/l, 0–0.12 µg/l, 0–0.24 µg/ml	+	(130)
Micronucleus *in vivo*, erythrocytes		−	(130)
Mouse lymphoma, L5178Y (TK+/TK−)	6.25–200 µg/l	+	(125)
UDS human cells	6.5–650 mg/ml	+	(104)
UDS rat hepatocytes	0.005–1.25 mg/ml	−	(131)
UDS mouse forestomach epithelium	1–100 mg/kg	−	(139)
Sex-linked recessive lethal gene mutation, *Drosophila melanogaster*		−	(140)
Sperm morphology, mouse		NC	(45, 46)
Dominant lethal test, rodents	With dose response	NC	(137)
Recombination assay, spot test, DNA effects, *Bacillus subtilis* (H17 vs. M45)		+	(79)
Carcinogenicity studies *in vivo*, non-human		NC	(141)
Carcinogenicity studies, mouse	0, 317, 635 mg/l in diet	−	(142)
Carcinogenicity studies, rat	0, 150, 318, 326, 635 mg/l in diet	−	(142)
Long-term carcinogenesis assay. Rat	0, 4, 8 mg/kg in corn oil for 105 weeks	+	(125)
Long-term carcinogenesis assay. Mouse	0, 10, 20 mg/kg in corn oil for 105 weeks	+	(125)
Long-term carcinogenesis assay. Rat	0, 0.1 mg in 0.2 ml water for 111 weeks	−	(143)
Long-term carcinogenesis assay. Mouse	0, 10, 20 mg/kg in corn oil for 104 weeks	+	(144)
Long-term carcinogenesis assay. Rat	0, 4, 8 mg/kg in corn oil for 104 weeks	+	(125)

**21. Dimetridazole (551-92-8)**			
*Salmonella typhimurium*, TA98, TA100, TA1535, TA1537, TA1538		+	(145)
*Salmonella typhimurium*, TA100		+	(146)
*Salmonella typhimurium*, TA97, TA98, TA100, TA102	50–200 µg/plate	+	(147)
Comet assay in human lymphocytes	354.3 mg/ml	+	(148)

**22. Fenbendazole (43210-67-9)**			
*Salmonella typhimurium* (none), TA100, TA97, TA98, TA102 (rat, liver, S-9, aroclor 1254), TA100, TA97, TA98, TA102.	5–1,000 µg/plate	−	(149)
Chromosomal damage in Chinese hamster lung (CHL) cells	0.78 mg/ml	+	(150)
Cytotoxicity to 10T1/2 cells	0.04–1.60 mg/ml	+	(150)
Morphological transformation in mouse embryo fibroblasts	0.08–0.4 mg/ml	+	(150)

**23. Fenchlorphos (299-84-3)**			
SCE, human somatic cells *in vitro*		+	(120)

**24. Fenthion (55-38-9)**			
Ames reverse gene mutation	0.1–20 µg/plate	−	(151)
*Bacillus subtilis* (H17 vs. M45)	20 µg/plate	−	(132)
*E. coli* polA (W3119 vs. P3478), recombination assay, DNA effects(bacterial DNA repair)		NC	(79)
*E. coli* WP2 uvra, tryptophan reverse gene mutation		−	(101)
SCE, non-human V79 cells *in vitro*		+	(152)
SCE, human somatic cells *in vitro*		NC	(120)
Mitotic recombination or gene conversion, *Saccharomyces cerevisiae*		−	(44)
*Bacillus subtilis* (H17 vs. M45), recombination assay, spot test, DNA effects (bacterial DNA repair)		NC	(79)
*Drosophila melanogaster*, sex-linked recessive lethal mutation		NC	(140)
UDS, human diploid fibroblasts *in vitro*		−	(66)
UDS, thymidine incorporation, rat hepatocytes	0, 5.0, 7.5, 10.0, 15.0, 30.0 µg/l	+	(153)
Chromosomal aberrations, CHO cells *in vitro*	0, 0.02, 0.04, 0.08, 0.15 µg/l	−	(153)
SCE *in vivo* and UDS *in vitro*		+	(154)
Chromosomal aberrations, human peripheral lymphocytes *in vitro*	0.5, 1.5, 2.5, 5.0 µg/ml	+	(151)
Long-term carcinogenesis assay. Mice	0, 0.1, 1, 5, 25 mg/l in diet for 2 years		(153)
Long-term carcinogenesis assay. Rats	0, 5, 20, 100 mg/l in diet for 2 years	−	(153)
Long-term carcinogenesis assay. B6C3F1 male mice	10 mg/l in diet for 103 weeks	+	(155)
Long-term carcinogenesis assay. B6C3F1 female mice	10 mg/l in diet for 103 weeks	−	(155)
Long-term carcinogenesis assay. F341 rat	200 mg/l in diet for 103 weeks	−	(155)

**25. Fenvalerate (51630-58-1)**			
*Salmonella typhimurium*, TA104	100–3,500 µg/plate	−	(156)
TA100	500–4,000 µg/plate	−	
TA97	100–4,000 µg/plate	−	
TA100	500–4,000 µg/plate	−	
TA98	100–3,000 µg/plate	−	
Micronuclei in bone marrow in mice *in vivo*	10, 20 mg/kg by i.p.	+	(157)
Peripheral blood for MN test	25, 50, 75, 100 mg/l	+	(98)
Chinese hamster V79 gene mutation	4–40 µg/l	−	(107)
Excision repairable DNA lesions		−	(99)
Chromosomal aberrations, Chinese hamster ovary (CHO-K1) *in vitro*	10, 25, 50, 100,150 µg/l	+	(158)
Long-term carcinogenesis assay. Rat (oral)	0, 1, 5, 25, 250 mg/l in diet for 2 years	−	(159)
Long-term carcinogenesis assay. Rat (oral)	1, 5, 25, 250, 1,000 mg/l in diet for 2 years	−	(160)
Long-term carcinogenesis assay. Mouse (oral)	0, 10, 50, 250, 1,250 mg/l in the diet for 2 years	−	(161)
Long-term carcinogenesis assay. Mouse (intragastric)	0, 40, 80 mg/kg in arachis oil for 120 weeks		(111)

**26. Fipronil (120068-37-3)**			
*Salmonella typhimurium*, TA98, TA100, TA1535, TA1537	0–0.5 mg/plate of 90.6% fipronil	+	(162)
Chromosomal aberrations, human lymphocytes *in vitro*	0, 4.69, 9.38, 18.75, 37.5, 75, 150, 300 μg/l	+	(162)
Sister chromatid exchanges (SCEs); DNA damage, comet assay *in vitro;* micronuclei (MN) in human peripheral blood lymphocytes	0.7,0.3 µg/l	+	(163)
Comet assay with gillsin, the fish Rhamdia Quelen; nuclear morphological alterations	0.05, 0.10, 0.23 µg/l	−	(164)
Micronucleus test in the Piscine	0.10, 0.23 µg/l	+	(164)
Chinese hamster V79 cells, HGPRT mutations	0, 0.8, 4, 20, 100, 500 µg/l	+	(162)
Bone marrow polychromatic erythrocytes, mouse micronucleus *in vivo*	0, 1, 5, 25 mg/kg b.w.	+	(162)
Long-term carcinogenesis assay. Rat (oral)	0, 0.5, 1.5, 30, 300 mg/l of 95.4% fipronil in diet for 104 weeks	+	(162)
Long-term carcinogenesis assay. Mouse (oral)	0, 0.1, 0.5, 10, 30 mg/l of 95.4% fipronil in diet for 78 weeks	+	(162)

**27. Flubendazole (31430-15-6)**			
*Salmonella typhimurium* (none), TA100, TA98; *Salmonella typhimurium* (rat, liver, S-9, aroclor 1254), TA98, TA100	0.01–10 µg/plate	−	(165)

**28. Furapromide (1951-56-0)**			
Chromosomal aberrations, V79 cells		+	(166)
*Salmonella typhimurium*, TA98, reverse mutation *Neurospora crassa*, forward gene mutation		+	(167)
			(168)
Chromosomal aberrations, V79, HPRT	10–120 µmol	+	(166)
*Saccharomyces cerevisiae*, mitotic recombination or gene conversion	7–567 µmol	+	(44)
*Salmonella typhimurium* (TA98, TA100, TA1535, TA1537, and TA1538)		+	(115, 116, 169)

**29. Furapyrimidone (75888-03-8)**			
*Salmonella typhimurium*, TA98, TA100; *Salmonella typhimurium*, TA98, TA100 (S-9), reverse mutation	0.01–10 µg/plate	+	(170)

**30. Imidacloprid (138261-41-3)**			
*Salmonella typhimurium*, TA100 (rat, Liver, S-9)	25–10 µg/plate	−	(171)
*Salmonella typhimurium*, TA98 (rat, Liver, with or without S-9)	25–100 µg/plate	+	(171)
*Salmonella typhimurium*, TA97, TA98, TA100, TA102 (S9)	40, 200, 1,000, 5,000 µg/plate	−	(172)
Micronuclei test in mouse bone marrow	23, 45, 90 mg/kg b.w.	−	(172)
Chromosome aberration in primary spermatocytes testicle	38, 75, 150 mg/kg b.w.	−	(172)
Micronucleus (MN) test *in vivo*, amphibian	165 mg/kg b.w.	+	(173)
Comet assay *in vivo*, amphibian	0.05, 0.1, 0.2, 0.5 mg/kg b.w.		
Bone marrow polychromatic erythrocytes in rats	100, 200, 300 mg/kg b.w.	+	(174)
Micronucleus *in vitro*, Human peripheral blood lymphocytes (rat, liver, S9)	0.2, 2, 20 µg/l	+	(175)
Micronuclei test in human peripheral lymphocytes SCE test in human peripheral lymphocytes	0.1, 0.5 mg/l	+	(176)
Comet assay, DNA damage, SCGE	0.05, 0.1, 0.2, 0.5 mg/l	+	(176)
Micronucleus (MN) formation in human lymphocytes *in vitro*	50 µg/l	+	(174)
SCE induction in human lymphocytes	Combination with metalaxyl at 100, 200 μg/l	+	(174)
SCE induction in human lymphocytes	0.1, 1, 5, 10, 50, 100 µg/l	−	(174)
Micronucleus in the rat bone marrow	200, 300, 400 mg/kg b.w.	+	(174)
DNA damage, Comet assay, SCGE		+	(177)
Micronucleus (MN) tests on *Hypsiboas pulchellus* tadpoles	25 mg/l for 96 h	+	(178)
DNA single-strand breaks on *Hypsiboas pulchellus* tadpoles	37.5 mg/l for 96 h	+	(178)
Nuclear abnormalities	12.5–37.5 mg/l	−	(178)
Chromosome abnormality on sperm deformity of the earthworm	0.2 mg/kg dry soil	+	(179)
DNA damage in human peripheral blood lymphocytes exposed *in vitro*		+	(180)
Long-term carcinogenesis assay. Rat (male)	0, 100, 300, 900, 1,800 mg/l	+	(181)
Long-term carcinogenesis assay. Mice	0, 100, 330, 1,000, 2,000 mg/l	−	(181)

**31. Ivermectin (70288-86-7)**			
Carcinogenicity studies, rats	0, 2 mg/l in diet for 1 year	−	(182)

**32. Lindane (58-89-9)**			
*Salmonella typhimurium, Serratia marcescens*, forward and reverse gene mutation, host-mediated assay		NC	(128)
MN-forming activity in MCF-7 and PC-3 cells	10^−12^, 2 × 10^−12^, 10^−11^, 2 × 10^−11^, 5 × 10^−11^ g/ml	+	(183)
Chromosomal aberrations in human peripheral lymphocytes *in vitro*		+	(184)
Micronucleus (MN) formation in bone marrow *in vivo*		+	(185)
Sex-linked recessive lethal gene mutation, *Drosophila melanogaster*		NC	(140)
Chromosome aberrations, *Allium cepa*		+	(136)
Chromosome aberrations, *Hordeum vulgare* (barley)		+	(186)
Chromosome aberrations, *Vicia faba*		+	(187)
Chromosome aberrations, Tradescantia species		+	(187)
Mitotic recombination or gene conversion, *Saccharomyces cerevisiae*		+	(44)
DNA damage and the risk for cancer on human tonsillar	0.5, 0.75, 1.0 mg/ml	+	(188)
Carcinogenicity studies in mouse	12.5, 25 and 50 ppm for 80 weeks	−	(189)
Carcinogenicity studies *in vivo*, non-human		+	(141)
Long-term carcinogenesis assay. AVy/AVy, AVy/a, A/a mouse	160 mg/kg/day	+	(190)
Long-term carcinogenesis assay. Rats	0, 0.05, 0.45, 4.5, 18.7 mg/kg/day (male) 0, 0.06, 0.57, 5.6, 23.1 mg/kg/day (female)	−	(54)

**33. Malathion (121-75-5)**			
*Salmonella typhimurium*, TA98, TA100, TA97A, TA102, TA1535, TA1537, reverse mutation	33–1,650, 80–400 mg/l	−	(191, 117)
*E. coli* WP2 uvra, tryptophan reverse gene mutation		−	(101)
Recombination assay, spot test, DNA effect (bacterial DNA repair)	NR		(79)
SCE, non-human, V79 cells *in vitro*	NR	+	(119)
SCE, human somatic cells *in vitro*	NR	+	(120)
Chromosome aberrations	NR	+	(186)
Micronuclei in bone marrow *in vivo* (mice)	2.5, 5, 10 mg/kg i.p. or p.o.	+	(157)
Chromosomal aberrations, mouse (injection)	400 mg/kg b.w.	+	(192)
Chromosomal aberrations, mouse (oral) bone marrow cells *in vivo*	240 mg/l for 4 or 8 weeks, 120 mg/l for 8 weeks		
Chromosomal aberrations, CHO cells *in vivo*	25, 50, 76 µg/l	−	(193)
Chromosomal aberrations (rat, liver S-9, aroclor1254), CHO cells *in vivo*	303, 352,402 µg/l	+	(193)
*Drosophila melanogaster*, sex-linked recessive lethal mutation		−	(140)
UDS, human diploid fibroblasts *in vitro*		−	(66)
Histidine reverse gene mutation, Ames assay		−	(146)
Mitotic recombination or gene conversion		−	(44)
Micronucleus test, chromosome aberrations		−	(194)
Micronucleus test, mice(oral) bone marrow cells *in vivo*	120, 240 mg/l in diet for 2 weeks	+	(192)
Micronucleus test, mice (injection) bone marrow cells *in vivo*	200, 300 mg/kg b.w.		(192)
Micronucleus test, human peripheral lymphocytes *in vivo*	20, 50, 75, 100 µg/l	+	(195)
Micronucleus test, rat peripheral blood lymphocytes *in vivo*	0, 25, 50, 100, 150 mg/kg b.w.	−	(196)
Micronucleus test, rat peripheral blood polychromatic and normochromatic erythrocytes *in vivo*	150 mg/kg b.w.	+	(196)
Long-term carcinogenesis assay. Rats	0, 2,000, 4,000 mg/l in diet	−	(84, 85)
Long-term carcinogenesis assay. Mice	0, 8,000, 16,000 mg/l in diet	−	(122, 123)
Carcinogenicity studies *in vivo*		−	(141)

**34. Mebendazole (31431-39-7)**			
*Salmonella typhimurium* (rat, liver, S-9, aroclor 1254), TA100, TA98	0.5–5, 0.5–5 µg/plate	−	(165)
	0.01–10 µg/plate	+ +	
*Salmonella typhimurium* (none), TA100, TA98	0.01–10 µg/plate	−	(165)
Forward and reverse gene mutation, body fluid assay, *Salmonella typhimurium*, host-mediated assay		+	(197)
Genotoxicity in a diploid mitotic recombination or gene mutation; genotoxicity in a haploid yeast reversion assay; gene conversion assay (strain D5 of *Saccharomyces cerevisiae*)		−	(198)

**35. Mefloquine (53230-10-7)**			
Bacterial mutation (Ames)		−	(47)
Cytogenetics *in vivo*		−	(47)
Long-term carcinogenesis assay, mice	30 mg/kg/day	−	(75, 199)

**36. Metronidazole (443-48-1)**			
*Salmonella typhimurium*, forward and reversegene mutation, host-mediated assay		+	(197)
*Salmonella typhimurium*, forward and reverse gene mutation, body fluid assay		+	(200)
*Salmonella typhimurium*, TA98, TA100, TA1535, TA1537, and TA1538		+	(201)
*Salmonella typhimurium*, TA100	25–1,000 µg/plate	+	(202)
	300 µg/plate	+	(203)
	50–200 µg/plate	+	(147)
	1–66 µg/plate	+	(204)
	50–12,800 µg/plate	+	(205)
*Salmonella typhimurium*, TA97, TA100, TA102, TA98	50–200 µg/plate	+	(147)
*Salmonella typhimurium*, TA1538, TA1537, TA100, TA98, TA1535		−	(206)
*E. coli*, none	0.01–0.5 mg/ml	+	(101)
	25–1,000 µg/l	+	(207)
*E. coli* (rat, liver, S-9, Aroclor 1254)	25–1,000 µg/l, 25–500 µg/l	−	(207)
*E. coli* WP2 uvra, Tryptophan reverse gene mutation	With dose response	+	(101)
Comet assay in human lymphocytes	292.1 mg/ml	+	(148)
Chromosome aberration (CA) *in vivo*	10, 20, 40 mg/kg b.w.	+	(208)
Micronucleus (MN) in the bone marrow cells of Balb/c mice *in vivo*	10, 20, 40 mg/kg b.w.	+	(208)
SCE *in vivo*, non-human		NC	(209)
SCE *in vitro*, human lymphocytes	With dose response	+	(210)
Micronucleus *in vivo*, bone marrow polychromatic lymphocytes	23, 70, 160 mg/kg b.w.	+	(211)
Mitotic recombination or gene conversion, *Saccharomyces cerevisiae*		−	(44)
Micronucleus test *in vivo*, chromosome aberrations, mammalian polychromatic erythrocytes		−	(43)
Chromosomal aberrations *in vitro*, human lymphocytes	0.1, 1, 10, 50 µg/l	+	(119)
Forward gene mutation, *Neurospora crassa*		+	(168)
Aneuploidy, chromosome aberrations, *Neurospora crassa*		−	(168)
*Neurospora crassa*, human		NC	(45, 46)
Sex-linked recessive lethal gene mutation, *Drosophila melanogaster*		NC	(140)
Carcinogenicity studies *in vivo*, non-human		+	(141)
UDS and cytogenetics *in vitro*		+	(47)
Carcinogenicity studies, mouse		+	(212)
Carcinogenicity studies, rat		+	(212)
Tumor promotion studies, mouse			(213)

**37. Niclosamide (50-65-7)**			
*Salmonella typhimurium* (none), TA1978, UTH8413, TA1538, TA98; *Salmonella typhimurium* (rat, liver, S-9, aroclor 1254), TA1978, UTH8413, TA1538, TA98	1–50 µg/plate	−	(74)
*Salmonella typhimurium* (rat, liver, S-9, aroclor 1254), TA98 (NR), YG1020, YG1021, YG1024	0.5–15 µg/plate	−	(74)
	0.5–20 µg/plate	+	(214)
SCE *in vitro*, Human lymphocytes		+	(215)

**38. Nitroscanate (19881-18-6)**			
*Salmonella typhimurium* (none), TA98, TA98(NR), TA98(1,8-Dnp6), TA100, TA100(NR), YG1024, YG1021, TA98, TA98(1,8-Dnp6), TA100	1–160 µg/plate	+	(216)
	20–160 µg/plate	−	
	20–320 µg/plate	+	
	10–80 µg/plate	+	
	10–80 µg/plate	−	
	0–9 µg/plate	+	
	0–40 µg/plate	+	
	10–320 µg/plate	+	
*Salmonella typhimurium* (rat, liver, S-9, aroclor 1254), TA98, TA98(NR), TA100, TA100(NR), TA98, TA98(1,8-Dnp6), TA100	10–160 µg/plate	+	(216)
	10–160 µg/plate	−	
	10–80 µg/plate	+	
	10–160 µg/plate	−	(216)
	10–320 µg/plate	+	

**39. Nitroxinil (1689-89-0)**			
*Salmonella typhimurium* (rat, liver, S-9, kanechlor 400), TA100, TA98, TA1535, TA1537, TA1538; *Salmonella typhimurium* (none), TA100, TA98, TA1535, TA1537, TA1538	0.05–5 mg/plate	−	(61)
Chromosomal aberrations *in vivo*, mouse bone marrow cells	0, 10, 20, 30, 40 mg/kg once	+	(80)
*Salmonella typhimurium* (none), TA1537	0–1,000 µg/plate	−	(56)
Micronucleus test *in vivo*, chromosome aberrations, mammalian polychromatic erythrocytes		−	(61)

**40. Oxfendazole (53716-50-0)**			
Chromosomal aberrations *in vivo*, spermatocytes and bone marrow cells	1,000 µg/kg	+	(217)

**41. Pentamidine (100-33-4)**
Salmonella typhimurium, TA98, TA100, reverse mutation; *Salmonella typhimurium*, TA98, TA100 (rat, liver S-9, Phenobarbital), reverse mutation	0.01–1 µmol/plate	−	(218)

**42. Permethrin (52645-53-1)**			
*Salmonella typhimurium*, TA98, TA100	100–3,000 µg/plate	−	(107)
*Salmonella typhimurium*, TA98, TA100	5–1,000 µg/plate	−	(219)
*Salmonella typhimurium*, TA98, TA100	1–20 mg/plate	−	(220)
*Salmonella typhimurium*, TA98, TA100, TA97A	39–2,730 mg/l	−	(191)
*Salmonella typhimurium*, TA1535, TA1537, TA98, TA100, *E. coli*	1–7,500 µg/plate	−	(62)
Chinese hamster V79, rat hepatocytes	4–40 µg/l	−	(107)
UDS *in vitro*, DNA effects, human diploid fibroblasts		−	(66)
Mitotic recombination or gene conversion, *Saccharomyces cerevisiae*		−	(44)

**43. Piperazine (110-85-0)**			
*Salmonella typhimurium* (none), TA100, TA1535, TA1537, TA98, TA100; *Salmonella typhimurium* (rat, liver, S-9, aroclor 1254), TA100, TA1535, TA1537, TA98; *Salmonella typhimurium* (hamster, liver, S-9, aroclor 1254), TA100, TA1535, TA1537, TA98	33–2,167 µg/plate	−	(221)
*Salmonella typhimurium* (rat, liver, S-9, aroclor 1254), TA100, TA1535, TA1537, TA98			
*Salmonella typhimurium* (hamster, liver, S-9, aroclor 1254), TA100, TA1535, TA1537, TA98			
*Salmonella typhimurium* (rat, liver, S-9, PCB), TA100, TA98			(222)

**44. Praziquantel (55268-74-1)**			
*Salmonella typhimurium* (TA98, TA100, TA1535, TA1537, TA1538)		−	(223)
*Salmonella typhimurium* (none), TA1537, TA1535, TA100, TA1538, TA98; *Salmonella typhimurium* (rat, liver, S-9, kanechlor 400), TA1537, TA1535, TA100, TA1538, TA98	0–1,000 µg/plate	−	(56)
*Salmonella typhimurium*, forward and reverse gene mutation, host-mediated assay		+	(197)
Forward and reverse gene mutation, body fluid assay, *Salmonella typhimurium*		NC	(224)
Forward gene mutation, Schizo saccharomyces pombe		−	(225)
Sex-linked recessive lethal gene mutation, *Drosophila melanogaster*		−	(140)
Mitotic recombination or gene conversion, *Saccharomyces cerevisiae*		−	(44)
Dominant lethal test, rodents		−	(226)
Carcinogenicity studies, Hamster	0, 300 mg/kg in corn oil for 40 weeks	−	(227)

**45. Pyrimethamine (58-14-10)**			
Bacterial mutation (Ames)		−	(47)
Micronucleus test (MN) bone marrow in mice *in vivo*	40 mg/kg b.w.	+	(228)
The transplacental MN test in mice *in vivo*	40 mg/kg b.w.	−	(228)
Cytogenetics *in vitro*		+	(47)
DNA damage on ICR mice (oral)	50 mg/kg b.w.	+	(229)
Embryonic and maternal genotoxicity	50 mg/kg b.w.	+	(229)
Cytogenetics *in vivo*		+	(47)
DNA damage, SCGE, Comet assay in mice and rats	50, 120 mg/kg b.w., respectively	+	(230)
MLA		+	(47)
Micronucleus assay *in vitro*, cultured human lymphocytes		−	(231)
Long-term carcinogenesis assay, B6C3F1 mice (female)	1,000 mg/l in diet	−	(75, 122, 123)
Long-term carcinogenesis assay, mice (lung tumors)	25 mg/kg i.p.	+	(75, 122, 123)
Long-term carcinogenesis assay, F344 rats	400 mg/l in diet	−	(75, 122, 123)

**46. Quinine (130-95-0)**			
*Salmonella typhimurium*, TA98, TA100, reverse mutation, *Salmonella typhimurium*, TA98, TA100 (rat, liver S-9)	20–50 µg/plate	−	(78)

**47. RH-5849 (112225-87-3)**			
*Salmonella typhimurium*, TA98, TA100, TA97A, TA102, TA100 reverse mutation	5, 50, 500, 5,000 µg/plate	−	(232)
Micronuclei test in mouse bone marrow *in vivo*	42, 84, 168 mg/kg b.w.	−	(232)
Chromosome aberration, primary spermatocytes of testis	50, 100, 200 mg/kg/d for 5days	−	(232)
Micronuclei test in human peripheral lymphocytes SCE test in human peripheral lymphocytes	25, 100 mg/l	+	(176)
Comet assay, DNA damage, SCGE	5, 25, 50, 100 mg/l	+	(176)
Chromosome abnormality on sperm deformity of the earthworm	100 mg/kg dry soil	+	(179)
Micronucleus(MN) test in human lymphocytes *in vitro*, Micronucleus(MN) test in rat bone marrow *in vivo*	50 mg/ml	+	(174)
	300 mg/kg b.w.		
SCE in human lymphocytes	100, 200 mg/ml	+	(174)
DNA strand breaks and DNA damage		+	(177)
Micronucleus(MN) test in mouse	23, 45, 90 mg/kg b.w.	−	(172)
Chromosome aberration Primary spermatocytes of testis	38, 75, 150 mg/kg b.w.	−	(172)

**48. Tetramethrin (7696-12-0)**			
*Salmonella typhimurium*, TA98; *Salmonella typhimurium*, TA98 (rat, liver S-9, polychlorinated biphenyl)	0.1–1 mg/plate	+	(220)
*Salmonella typhimurium*, TA100; *Salmonella typhimurium*, TA100 (rat, liver S-9, polychlorinated biphenyl)	0.1–1 mg/plate	+	
*Salmonella typhimurium*, TA98, TA100; *Salmonella typhimurium*, TA98, TA100 (S9)	5–1,000 µg/plate	−	(219)

**49. Thiophanate (23564-05-8)**			
*Salmonella typhimurium* (none), TA100, TA98, TA1535, TA1537, TA97	33–10,000 µg/plate	−	(204)
*Salmonella typhimurium* [hamster, liver, S-9, aroclor 1254 (10% or 30%)], TA100, TA1535, TA97, TA98, TA100; *Salmonella typhimurium* [liver, S-9, aroclor 1254 (10 or 30%)], TA100, TA1535, TA97, TA98, TA1537	100–10,000 µg/plate	−	(204)
Chromosome aberrations, aneuploidy, *Aspergillus nidulans*		+	(233)
Chromosome aberrations *in vivo*, mammalian germ cells		−	(40)

**50. Tiabendazole (148-79-8)**			
*Salmonella Typhimurium* (none), TA100, TA98; *Salmonella typhimurium* (hamster, liver, S-9, aroclor 1254, 30%), TA100, TA98; *Salmonella typhimurium* (rat, liver, S-9, aroclor 1254, 30%), TA100, TA98	100–10,000 µg/plate	−	(60)
*Salmonella typhimurium* (hamster, liver, S-9, aroclor 1254, 10%), TA98	100–10,000 µg/plate	+	(60)
*Salmonella typhimurium* (none), TA98, TA100, TA97, TA104, *E. coli*, WP2S/PKM101	50–400 µg/l	+	(234)
Micronucleus test *in vivo*, chromosome aberrations, mammalian polychromatic erythrocytes		+	(235)
Mitotic recombination, *Aspergillus nidulans*		NC	(233)
Chromosome aberrations, *Aspergillus nidulans*, aneuploidy		+	(233)
Micronucleus (none) *in vitro*, V79 cells	0.5–700 µg/l	+	(236)
Micronucleus (none) *in vitro*, human lymphoblastoid wtk1 cells	0, 50, 100, 200 µg/l	+	(234)
Carcinogenicity studies, mouse	0, 0.8, 1.2, 1.6% in diet for 44 weeks	−	(237)
	0, 0.031, 0.125, 0.5% in diet for 78 weeks		
Long-term carcinogenesis assay, rats	0, 0.05, 0.1, 0.2, 0.4% in diet for 104 weeks	−	(238)

**51. Tinidazole (19387-91-8)**			
*Salmonella typhimurium*, TA100, reverse mutation	10–100 µg/plate	+	(239)
*Salmonella typhimurium*, TA100 (rat, liver S-9, aroclor 1254), reverse mutation	10–100 µg/plate	−	(239)
*Salmonella typhimurium*, TA98, reverse mutation	10–100 µg/plate	+	(239)
*Salmonella typhimurium*, TA98 (rat, liver S-9, aroclor 1254), reverse mutation	10–800 µg/plate	+	(205)
*Salmonella typhimurium*, UTH8414, reverse mutation	50–12,800 nmol/plate	+	
*Salmonella typhimurium*, TA98, TA100, reverse mutation	50–3,200 nmol/plate	+		+
*Salmonella typhimurium*, TA100, reverse mutation				
*Salmonella typhimurium*, TA100(1,8-DNP6),YG1029, TA100 (NR), reverse mutation				
*Salmonella typhimurium*, TA100 (NR), TA100 (rat, liver S-9, aroclor 1254), reverse mutation				

**52. Triclabendazole (68786-66-3)**			
Chromosomal aberrations *in vitro*, river buffalo lymphocytes	25, 50, 100 µg/l	+	(239)
Micronucleus *in vitro*, river buffalo Lymphocytes, micronucleus formation in lymphocyte cultures of the river buffalo	25, 50, 100 µg/l	+	(239)
SCEs in lymphocyte cultures of the river buffalo	25, 50, 100 µg/ml		

*The name of each drug is followed by the CAS number. For each type of assay: “+,” positive response; “−,” negative response; NR, not reported; NT, not tested; p.o., oral; i.p., intraperitoneal; UDS, DNA repair synthesis; MLA, gene mutation, mouse lymphoma L5178Y cells, TK locus; HGPRT, gene mutation, hgprt locus; SCE, sister chromatid exchange; MN, micronucleus; Trans., cell transformation;HID, highest ineffective dose; LED, lowest effective dose*.

*Pharmaceuticals without retrievable data: Amicarbalide, Abamectin, Acetarsone, Amprolium, Arecoline Hydrobromide, Artemether, Artemisinine, Artesunate, Avermectin, Azamethiphos, Amprolium Hydrochloride, Bunamidine, Carbarsone, Chiniofon, Clopidol, Clorsulon, Closantel Sodium, Cyromazine, Destomycin A, Diamphenethide, Diclazuril, Diethylcarbamazine, Diethylcarbamazine, Dihydroartemisinin, Diiodohydroxyquinoline, Diloxanide, Diminazene, Dinitolmide, Dithiazanine Iodide, Doramectin, Emetine, Epsiprantel, Ethopabate, Febantel, Fexinidazole, Fluvalinate, Hainanmycin, Halofuginone, Haloxon, Hetolin, Hexachloroparaxylene, Hydroxychloroquine, Hygromycin B, Imidocarb, Dipropionate, Isometamidium, Levamisole, Lumefantrine, Maduramicin, Malaridine, Metrifonate, Milbemycin Oxime, Monensin Sodium, Morantel, Moxidectin, Naftalofos, Naphthalophos, Nicarbazin, Nitazoxanide, Nitroquine, Oxantel, Oxibendazole, Oxinothiophos, Phanquinone, Phoxim, Piperanitrozole, Piperaquine, Primaquine, Propetamphos, Pyramine, Pyrantel, Quinapyramine, Rafoxanide, Resorantel, Robenidine, Salinomycin, Secnidazole, Semduramicin, Sodium stibogluconate, Sulfaquinoxaline, Sulfur Sublimat, Tetramisole, Thiacetarsamide, and Toltrazuril*.

Table 4 summarizes the total number of antiparasitics and the following are included: the number of antiparasitics with at least one genotoxicity or carcinogenicity test result and with data required by the present guidelines; the number of antiparasitics only tested for genotoxicity or carcinogenicity. It also presents the antiparasitics with results in *in vitro* data required by present guidelines; the number of antiparasitics that have at least one result in long-term carcinogenesis assays in rats or mice; and the number of antiparasitics in genotoxicity assays (bacterial mutagenicity, *in vitro* tests for gene mutation and for chromosomal damage, *in vivo* cytogenetic tests, and other types of genotoxicity assays). Of 136 antiparasitics examined, 52 (38.2%) had at least one genotoxicity or carcinogenicity test result, and 32 (23.5%) were tested only for either genotoxicity or carcinogenicity. Among 20 antiparasitics with results available for both genotoxicity and carcinogenicity, 16 had all the results required by the present guidelines for testing of pharmaceuticals: 8 of them—Albendazole, Coumaphos, Cypermethrin, Deltamethrin, Diazinon, Fenvalerate, Malathion and Tiabendazole—tested positive in genotoxicity assays but gave at least one negative result in carcinogenesis assays; 8 antiparasitics (Chlordimeform, Dichlorvos, Fenthion, Fipronil, Lindane, Metronidazole, Pyrimethamine, and Imidacloprid) gave positive responses in both genotoxicity and carcinogenicity. The remaining four with both genotoxicity and carcinogenicity data were not in agreement with the current guidelines: Amitraz and Praziquantel gave positive responses in genotoxicity but were non-carcinogenic; Atovaquone tested negative in genotoxicity but positive in mouse carcinogenicity; and Mefloquine produced negative responses in both genotoxicity and carcinogenicity.

**Table 4:** Overview of genotoxicity and carcinogenicity testing of antiparasitics.

Antiparasitics with at least one genotoxicity or carcinogenicity tests results (Table 3)	52 (38.2%)^a^
Antiparasitics without retrievable genotoxicity or carcinogenicity data	84 (61.8%)
Antiparasitics with all genotoxicity and carcinogenicity data required by present guidelines (Table 3: 2, 10, 13, 15, 17, 19, 20, 24–26, 30, 32, 33, 36, 45, 50)^b^	16 (11.8%)
Antiparasitics tested not according to present guidelines	36 (26.5%)
Antiparasitics with least one genotoxicity and carcinogenicity test results (Table 3: 2, 3, 7, 10, 13, 15, 17, 19, 20, 24–26, 30, 32, 33, 35, 36, 44, 45, 50)	20 (14.7%)
Antiparasitics tested only for genotoxicity (Table 3: 1, 4–6, 8, 9, 11, 12, 14, 16, 18, 21–23, 27–29, 34, 37–43, 46–49, 51, 52)	31 (22.8%)
Antiparasitics tested only for carcinogenicity (Table 3: 31)	1 (0.7%)
Antiparasitics with at least one results in tests for bacterial mutagenicity (Table 3: 1–11, 13–22, 24–30, 32–39, 41–51)	47 (34.6%)
Antiparasitics with at least one results in tests for gene mutation in mammalian cells (Table 3: 1, 6, 7, 10, 17, 19, 20, 24–26, 28, 32–34, 36, 44, 45, 50)	18 (13.2%)
Antiparasitics with at least one results in *in vitro* tests for SCE, chromosomal aberrations, aneuploidy, or micronucleus in animal or human cells (Table 3: 6, 7, 9, 10, 13–26, 28, 30, 32, 33, 36, 37, 42, 45, 47, 49, 50, 52)	33 (24.3%)
Antiparasitics with results in *in vitro* data required by present guidelines (Table 3: 1–3, 13, 17–19, 24–26, 28, 30, 42, 50, 52)	15 (11.0%)
Antiparasitics with at least one results in *in vivo* tests for SCE, chromosomal aberrations, or micronucleus in animal or human cells (Table 3: 6–20, 24–26, 30, 32, 33, 35, 36, 39, 40, 45, 47, 49, 50)	31 (22.8%)
Antiparasitics which underwent testing for DNA damage or DNA repair synthesis (Table 3: 3, 10, 11, 14–16, 18–21, 24, 25, 30, 32, 33, 36, 42, 45, 47)	19 (14.0%)
Antiparasitics which underwent testing in other types of genotoxicity assays (Table 3: 1, 10, 15, 19, 20, 22, 24, 26, 28, 30, 32–34, 36, 42, 44, 45)	17 (12.5%)
Antiparasitics examined for genotoxicity in human cells (Table 3: 2, 6, 14–16, 19–21,23, 24, 26, 30, 32, 33, 36, 37, 42, 45, 47, 50)	20 (14.7%)
Antiparasitics tested for carcinogenicity in mice (Table 3: 2, 3, 7, 10, 13, 15, 17, 19, 20, 24–26, 30, 32, 33, 35, 36, 45, 50)	19 (14.0%)
Antiparasitics tested for carcinogenicity in rats (Table 3: 2, 3, 7, 10, 13, 15, 17, 19, 20, 24–26, 30–33, 35, 36, 45, 50)	20 (14.7%)
Antiparasitics tested for carcinogenicity in both mice and rats (Table 3: 2, 3, 7, 10, 13, 15, 17, 19, 20, 24–26, 30, 32, 33, 35, 36, 45, 50)	19 (14.0%)
Antiparasitics tested for carcinogenicity in other species (Table 3: 44)	1 (0.7%)

*^a^Values in parentheses indicate the percentage of the 136 antiparasitics considered*.

*^b^Number and percentage in parentheses are those of antiparasitics of Table 3*.

Additional 32 antiparasitics were only tested in either genotoxicity or carcinogenicity. Only one (Ivermectin) had retrievable results in carcinogenicity. As for the rest, 31 antiparasitics had the data of genotoxicity. Twenty-one antiparasitics (Acriflavine, Closantel, Chloroquine, Cyfluthrin, Danex, Diaveridine, Dimetridazole, Fenbendazole, Fenchlorphos, Furapyrimidone, Furapromide, Mebendazole, Nitroscanate, Nitroxinil, Niclosamide, Oxfendazole, RH-5849, Tetramethrin, Thiophanate, Tinidazole, and Triclabendazole) gave positive responses in at least one genotoxicity assay; 10 antiparasitics (Amodiaquine, Amoscanate, Amphotericin B, Bithionol, Bromofenofos, Flubendazole, Pentamidine, Permethrin, Piperazine, and Quinine) were found to be negative in all the considered genotoxicity assays. With regard to the different types of genotoxicity assays: there were 47 antiparasitics with at least one result in tests for bacterial mutagenicity; 18 antiparasitics with at least one result in tests for gene mutation in mammalian cells; 33 antiparasitics in *in vitro* tests for SCE, chromosomal aberrations, aneuploidy, or micronucleus in animal or human cells; 15 antiparasitics with results in *in vitro* data required by present guidelines; 31 antiparasitics in *in vivo* tests for SCE, chromosomal aberrations, or micronucleus in animal or human cells; 19 antiparasitics in DNA damage or DNA repair synthesis; 17 antiparasitics in other types of genotoxicity assays; and 20 antiparasitics examined for genotoxicity in human cells. With respect to carcinogenesis assays, 19 and 20 antiparasitics were tested for carcinogenicity in mice and rats, respectively. Among the antiparasitics with both the genotoxicity and carcinogenicity data, 19 antiparasitics tested for carcinogenicity in both mice and rats and only 1 in hamsters.

Table 5 provides the number of antiparasitics tested for each type of assay, including the genotoxicity and carcinogenicity studies. The results are indicated as positive, negative and discordant. When carcinogenicity testing is considered, 57.9% of antiparasitics were tested negative in mice, and 73.7% in rats. Five antiparasitics (nos. 7, 10, 26, 32, and 36) and three antiparasitics (nos. 10, 26, and 36) were carcinogenic in mice and rats, respectively. The percentage of concordant results in carcinogenicity assays between mice and rats is 85.7% (12 out of 14) and only 2 (nos. 7 and 32) antiparasitics have discordant results: no. 32 tested positive in mice and negative in rats, while no. 7 produced the opposite result. The occurrence of discordant results between mice and rats may be the differences in species (e.g., metabolic enzymes). Ten antiparasitics were in IARC of 2B and 3 ground classifications of carcinogens: Chloroquine, Danex, and Permethrin do not have available carcinogenicity data; Deltamethrin, Fenvalerate, and Malathion tested negative in rodents while positive results were given by Chlordimeform and Metronidazole. Dichlorvos (DDVP) and Pyrimethamine have discordant results of carcinogenicity in mice and rats. To interpret the tumor findings in a carcinogenicity study and provide a perspective on the relevance of rodents to human, the mechanism and some investigations in tumor profile (trans-species, trans-sex, and multisite *versus* single species, single sex, and single site) were suggested by the guidelines (15).

**Table 5:** Summary per assays type of antiparasitics with positive, negative, and discordant results.

Bacterial mutagenitity	Positive	4 (8.5%) (Table 3: 21, 26, 28, 29)
	Negative	29 (61.7%) (Table 3: 2–10, 13–15, 17, 19, 22, 24, 25, 27, 32, 33, 35, 39, 41–47, 49)
	Discordant	14 (29.8%) (Table 3: 1, 11, 16, 18, 20, 30, 34, 36–38, 44, 48, 50, 51)

Gene mutation in cultured mammalian cells	Positive	7 (38.9%) (Table 3: 1, 20, 26, 28, 32, 45, 50)
	Negative	11 (61.1%) (Table 3: 6, 7, 10, 19, 24, 25, 33, 34, 36, 42, 44)
	Discordant	0

*In vitro* cytogenetics	Positive	18 (54.5%) (Table 3: 1, 15, 16, 18, 19, 21, 22, 23, 26, 28, 32, 33, 36, 37, 45, 47, 50, 52)
	Negative	7 (21.2%) (Table 3: 3, 6, 7, 9, 13, 34, 42)
	Discordant	8 (24.2%) (Table 3: 2, 10, 14, 17, 20, 24, 25, 30)

*In vivo* cytogenetics	Positive	13 (41.9%) (Table 3: 11–15, 17, 19, 24–26, 32, 40, 50)
	Negative	8 (25.8%) (Table 3: 6–9, 18, 20, 35, 49)
	Discordant	10 (32.3%) (Table 3: 1, 2, 10, 16, 30, 33, 36, 39, 45, 47)

DNA lesions (*in vitro* and *in vivo*)	Positive	9 (47.4%) (Table 3: 3, 14, 16, 21, 30, 32, 36, 45, 47)
	Negative	3 (15.8%) (Table 3: 25, 33, 42)
	Discordant	7 (36.8%) (Table 3: 10, 15, 18–20, 24, 26)

Carcinogenesis in mice	Positive	5 (26.3%) (Table 3: 7, 10, 26, 32, 36)
	Negative	11 (57.9%) (Table 3: 2, 3, 13, 15, 17, 19, 22, 25, 30, 33, 35)
	Discordant	3 (15.8%) (Table 3: 20, 24, 45)

Carcinogenesis in rats	Positive	3 (15.8%) (Table 3: 10, 26, 36)
	Negative	14 (73.7%) (Table 3: 2, 3, 7, 13, 15, 17, 24, 25, 31–33, 35, 45, 50)
	Discordant	2 (10.5%) (Table 3: 20, 30)

Carcinogenesis in mice and rats	Discordant	2 (14.3%) (Table 3: 7, 32)

Carcinogenesis in mice and rats	Concordant	12 (85.7%) (Table 3: 2, 3, 10, 13, 15, 17, 25, 26, 33, 35, 36, 50)

*The antiparasitic was considered as positive when it gave only positive results and as negative when it gave only negative or inconclusive results. Discordant indicates the number of antiparasitics, of which the results of genotoxicity assays were both positive and negative or inconclusive and he results of carcinogenicity assays performed in the same species were carcinogenic to mice or rats but not to rats or mice. In parentheses is the number of drugs in Table 3*.

### Re-Evaluation of *In Vitro* Genotoxicity Results

Table 6 presents the incidence of misleading positive effects in *in vitro* cytogenicity when using the reduction in a top dose of 1 mM. Of 33 antiparasitics with at least one result in *in vitro* tests for SCE, chromosomal aberrations, or micronucleus in animal or human cells, 25 (75.8%) antiparasitics had at least one retrievable dose in *in vitro* cytogenicity assays, while 8 (24.2%) antiparasitics had no available dose. Under the current *in vitro* genotoxicity testing guidelines for dose limits, 10 (nos. 10, 14, 15, 16, 20, 21, 22, 32, 36, and 47) antiparasitics were identified as genotoxins at dose levels more than 1 mM. The re-evaluation results indicated the misleading positive response in the previous reports. Fifteen (nos. 1, 2, 3, 13, 17, 18, 19, 24, 25, 26, 28, 30, 42, 50, and 52) antiparasitics had *in vitro* genotoxicity results consistent with ICH S2 (R1).

**Table 6:** Re-evaluate the *in vitro* cytogenetic results according to the ICH S2 (R1).

Test system (*in vitro* cytogenetic assays)	Dose or concentration (LED or HID)	Result	Conversion unit (mM)	ICH S2 (R1), 1 mM Concordant
**1. Acriflavine (1) (259.70)**				
CHO, CHO-K1-BH4 (HGPRT)	0.5–4 µg/l	+	1.54 × 10^−5^	Y

**2. Albendazole (2) (265.33)**				
MN, peripheral blood lymphocytes	10–100 µg/ml	+	0.377	Y
MN, human lymphocytes	10–100 µg/ml	+	0.377	Y

**3. Amitraz (3) (293.23)**				
DNA damage on hamster cells, comet assay	3.75 µg/l	+	1.28 × 10^−5^	Y

**4. Chlordimeform (10) (196.68)**				
DNA effects (human diploid fibroblasts FL cell)	10^−6^ to 10^−3^ g/ml	+	5.08	N

**5. Coumaphos (13) (362.78)**				
CA *in vitro*, CHO cells (rat, liver, S-9, aroclor 1254)	100, 300, 1,000 µg/l	−	2.76 × 10^−3^	Y
CA *in vitro*, CHO cells (none)	99.5, 299, 995 µg/l	−	2.7 × 10^−3^	Y

**6. Cyfluthrin (14) (434.29)**				
CA, human peripheral blood lymphocytes	1,000, 2,000 mg/ml	+	4.61 × 10^3^	N
SCE, human peripheral blood lymphocytes	500, 1,000, 2,000 mg/ml	−	4.61 × 10^3^	N
MN, human peripheral blood lymphocytes	500, 1,000, 2,000 mg/ml	+	4.61 × 10^3^	N
DNA damage, epithelial cells of human nasal mucosa	0.05, 0.1, 0.5, 0.75, 1.0 mg/ml	+	2.303	N
DNA damage and comet assay in fish	5.6 mg/l beta-cyfluthrin for 48 h	+	1.29 × 10^−2^	Y
CA *in vitro*	500, 1,000, 2,000 µg/l	−	4.61 × 10^−3^	Y
SCE in blood lymphocytes	500, 1,000, 2,000 µg/l	−	4.61 × 10^−3^	Y
Mouse bone marrow cells *in vitro*	1,000 µg/l	+	2.30 × 10^−3^	Y

**7. Cypermethrin (15) (416.32)**				
CAs, human peripheral lymphocytes	5, 10, 15, 20 mg/ml	+	48.0	N
SCE, human peripheral lymphocytes	5, 10, 15, 20 mg/ml	+	48.0	N
MN, human peripheral lymphocytes	5, 10 mg/ml	+	24.0	N
CA in highly mitotic kidney cells	0.4, 0.8,1.2 µg/l for 48 and 72 h	+	2.88 × 10^−6^	Y
MN, erythrocytes of a freshwater fish	0.4, 0.8,1.2 µg/l for 48 and 72 h	+	2.88 × 10^−6^	Y
Peripheral blood for MN test	20, 30, 40, 50 mg/l	+	0.120	Y

**8. Danex (16) (257.45)**				
UDS human cells	0.4–4,000 mmol	+	4.0 × 10^3^	N
CA, V79 cell	0.04–0.8 mmol	−	8.0 × 10^2^	N

**9. Deltamethrin (17) (505.20)**				
CA, CHO cells *in vitro*	0, 19, 38, 75, 150 µg/l	+	2.97 × 10^−4^	Y
V79/6-thioguanine, Chinese hamater V79	4–40 µg/l	−	7.92 × 10^−5^	Y

**10. Diaveridine (18) (260.29)**				
CA in cultured CHL cells	12.5, 25, 50, 100 µg/l	+	3.84 × 10^−4^	Y
CA	100 µg/l,48 h	+	3.84 × 10^−4^	Y

**11. Diazinon (19) (304.35)**				
DNA damage, human blood lymphocytes	750 µg/l	+	2.46 × 10^−3^	Y

**12. Dichlorvos(DDVP) (20) (220.98)**				
CA *in vitro*, CHO cells	16, 50, 100, 160 µg/l	+	7.24 × 10^−4^	Y
	50, 160, 500, 1,600 µg/l	+	7.24 × 10^−3^	Y
	500, 750, 1,000 µg/l	+	4.53 × 10^−3^	Y
CA, V79	1.25–5 µg/l	−	2.26 × 10^−5^	Y
CHO, CHO-k1-bh4 (HGPRT)/6-thioguanine	50–150 µg/l	+	6.79 × 10^−4^	Y
Mouse lymphoma, L5178Y (TK+/TK−)	0–0.33 µg/l, 0–0.12 µg/l,	+	1.49 × 10^−6^	Y
	0–0.24 µg/ml	+	1.09 × 10^−3^	Y
Mouse lymphoma, L5178Y (TK+/TK−)	6.25–200 µg/l	+	9.05 × 10^−4^	Y
UDS human cells	6.5–650 mg/ml	+	2.94 × 10^3^	N
UDS rat hepatocytes	0.005–1.25 mg/ml	−	5.66	N

**13. Dimetridazole (21) (141.12)**				
Comet assay, human lymphocytes	354.3 mg/ml	+	2.51 × 10^3^	N

**14. Fenbendazole (22) (299.34)**				
Chromosomal damage in CHL cells	0.78 mg/ml	+	2.61	N
Cytotoxicity to 10T1/2 cells	0.04–1.60 mg/ml	+	5.35	N
Morphological transformation in mouse embryo fibroblasts	0.08–0.4 mg/ml	+	1.34	N

**15. Fenthion (24) (278.33)**				
UDS, thymidine incorporation, rat hepatocytes	0, 5.0, 7.5, 10.0, 15.0, 30.0 µg/l	+	1.08 × 10^−4^	Y
CA, CHO cells *in vitro*	0, 0.02, 0.04, 0.08, 0.15 µg/l	−	5.39 × 10^−7^	Y
CA, human peripheral lymphocytes	0.5, 1.5, 2.5, 5.0 µg/ml	+	1.80 × 10^−2^	Y

**16. Fenvalerate (25) (419.90)**				
Peripheral blood for MN test	25, 50, 75, 100 mg/l	+	0.238	Y
Chinese hamster V79 gene mutation	4–40 µg/l	−	9.53 × 10^−5^	Y
CA, CHO-K1, *in vitro*	10, 25, 50, 100,150 µg/l	+	3.57 × 10^−4^	Y
CA, CHO-K1, *in vitro*	5, 10, 25, 50 µg/l	+	1.19 × 10^−4^	Y

**17. Fipronil (26) (437.20)**				
CA, human lymphocytes *in vitro*	0, 4.69, 9.38, 18.75, 37.5, 75, 150, 300 µg/l	+	6.86 × 10^−4^	Y
SCEs, DNA damage, comet assay	0.3,0.7 µg/l	+	1.60 × 10^−6^	Y
MN, human peripheral blood lymphocytes	0.3, 0.7 µg/l	+	1.60 × 10^−6^	Y
Comet assay with gillsin, the fish *Rhamdia quelen*	0.05, 0.10, 0.23 µg/l	−	5.26 × 10^−7^	Y
Nuclear morphological alterations	0.05, 0.10, 0.23 µg/l	−	5.26 × 10^−7^	Y
CA, V79 cells, HGPRT mutations	0, 0.8, 4, 20, 100, 500 µg/l	+	1.14 × 10^−3^	Y

**18. Furapromide (28) (224.22)**				
CA, V79 cell	10–120 µmol	+	1.20 × 10^−4^	Y

**19. Imidacloprid (30) (255.70)**				
MN, human peripheral blood lymphocytes	0.2, 2, 20 µg/l	+	7.82 × 10^−5^	Y
MN, human peripheral lymphocytes	0.1, 0.5 mg/l	+	1.96 × 10^−3^	Y
SCE, human peripheral lymphocytes	0.1, 0.5 mg/l	+	1.96 × 10^−3^	Y
Comet assay, DNA damage, SCGE	0.05, 0.1, 0.2, 0.5 mg/l	+	1.96 × 10^−3^	Y
MN, Human lymphocytes *in vitro*	50 µg/l	+	1.96 × 10^−4^	Y
SCE in human lymphocytes	Combination with metalaxyl at 100, 200 µg/l	+	7.82 × 10^−4^	Y
SCE induction in human lymphocytes	0.1, 1, 5, 10, 50, 100 µg/l	−	3.91 × 10^−4^	Y

**20. Lindane (32) (290.82)**				
Comet-forming activity in MCF-7 cells	10^−4^ g/ml	+	3.44	N
DNA damage and the risk for cancer on human tonsillar	0.5, 0.75, 1.0 mg/ml	+	34.4	N

**21. Metronidazole (36) (171.16)**				
Comet assay in human lymphocytes	292.1 mg/ml	+	1.71 × 10^3^	N
CA *in vitro*, human lymphocytes	0.1, 1, 10, 50 µg/l	+	2.92 × 10^−4^	Y

**22. Permethrin (42) (391.28)**				
Chinese hamster V79, rat hepatocytes	4–40 µg/l	−	1.02 × 10^−4^	Y

**23. RH-5849 (47) (296.40)**				
MN, human peripheral lymphocytes	25, 100 mg/l	+	0.337	Y
SCE, human peripheral lymphocytes	25, 100 mg/l	+	0.337	Y
Comet assay, DNA damage, SCGE	5, 25, 50, 100 mg/l	+	0.337	Y
MN, human lymphocytes *in vitro*	50 mg/ml	+	1.69 × 10^2^	N
SCE, human lymphocytes	100, 200 mg/ml	+	6.75 × 10^2^	N

**24. Tiabendazole (50) (210.19)**				
MN (none) *in vitro*, V79 cells	0.5–700 µg/l	+	3.33 × 10^−3^	Y
MN, human lymphoblastoid wtk1 cells	0, 50, 100, 200 µg/l	+	9.52 × 10^−4^	Y

**25. Triclabendazole (52) (359.66)**				
CA *in vitro*, river buffalo lymphocytes	25, 50, 100 µg/l	+	2.78 × 10^−4^	Y
MN *in vitro*, river buffalo lymphocytes	25, 50, 100 µg/l	+	2.78 × 10^−4^	Y
CA in lymphocyte	25, 50, 100 µg/l	+	2.78 × 10^−4^	Y
SCEs in lymphocyte	25, 50, 100 µg/ml	+	0.278	Y
MN in lymphocyte	25, 50, 100 µg/ml	+	0.278	Y

*The name of each antiparasitic is followed by the number in the Table 1 and molecular weight. For each type of assay: “+,” positive response; “−,” negative response; “Y,” consistent with results of the current guideline of ICH S2 (R1); “N,” discordant with results of the current guideline of ICH S2 (R1); UDS, DNA repair synthesis; MN, micronucleus; MLA, gene mutation, mouse lymphoma L5178Y cells, TK locus; HGPRT, gene mutation, hgprt locus; SCE, sister chromatid exchange; Trans., cell transformation; HID, highest ineffective dose; LED, lowest effective dose; CHO, Chinese hamster ovary; CHL, Chinese hamster lung. Pharmaceuticals with *in vitro* cytogenetic results but without the retrievable dose: Amphotericin B, Atovaquone, Bromofenofos, Fenchlorphos, Malathion, Niclosamide, Pyrimethamine, Thiophanate*.

### Correlation between the Genotoxicity Assays

Table 7 provides the correlation among the different types of genotoxicity assays of antiparasitics, the numbers and percentages of antiparasitics that tested concordant and discordant between each other. On the whole, the degree of coincident correlation was higher than the discordant results, which ranged from 84.6% between bacterial mutagenicity and gene mutation in mammalian cells to 55.6% between gene mutation in mammalian cells and *in vivo* cytogenetics. When bacterial mutagenicity was compared with the following assays: gene mutation in mammalian cells, *in vitro* cytogenetics, *in vivo* cytogenetics and DNA lesions, 13 (nos. 3, 14, 15, 17, 19, 22, 24, 25, 32, 33, 45, 47, and 49) antiparasitics gave negative results in bacterial mutagenicity. Among these antiparasitics, there were 2 (nos. 32 and 45), 8 (nos. 15, 22, 24, 25, 32, 33, 47, and 49), 7 (nos. 14, 15, 17, 19, 24, 25, and 32) and 5 (nos. 3, 14, 32, 45, and 47) antiparasitics that tested positive in gene mutation in mammalian cells, *in vitro* cytogenetics, *in vivo* cytogenetics and DNA lesions, respectively.

**Table 7:** Correlation between the results of genotoxicity assays of antiparasitics.

Couples of assays considered	No. of drugs with	
	Concordant results	Discordant results
Bacterial mutagenicity—gene mutation in mammalian cells	11 (84.6%) (6, 7, 10, 19, 20, 24–26, 28, 33, 42)	2 (16.7%) (32, 45)
Bacterial mutagenicity—*in vitro* cytogenetics	12 (60.0%) (3, 6, 7, 10, 13, 16, 20, 26, 28, 30, 36, 42)	8 (40.0%) (15, 19, 22, 25, 32, 33, 47, 49)
Bacterial mutagenicity—*in vivo* cytogenetics	11 (57.9%) (1, 6–9, 16, 26, 30, 35, 36, 49)	8 (42.1%) (13–15, 17, 19, 24, 25, 32)
Bacterial mutagenicity—DNA lesions	7 (58.3%) (16, 18, 20, 21, 25, 33, 42)	5 (41.7%) (3, 14, 32, 45, 47)
Gene mutation in mammalian cells—*in vitro* cytogenetics	9 (75.0%) (1, 6, 7, 10, 26, 28, 32, 42, 50)	3 (25.0%) (19, 25, 33)
Gene mutation in mammalian cells—*in vivo* cytogenetics	5 (55.6%) (6, 7, 26, 32, 50)	4 (44.4%) (19, 20, 24, 25)
Gene mutation in mammalian cells—DNA lesions	5 (83.3%) (25, 32, 33, 42, 45)	1 (16.7%) (36)
*in vitro* cytogenetics—*in vivo* cytogenetics	13 (81.2%) (2, 6, 7, 15, 16, 19, 25, 26, 30, 32, 36, 45, 50)	3 (18.8%) (13, 18, 49)
DNA lesions—*in vitro* cytogenetics	6 (66.7%) (16, 20, 24, 32, 42, 47)	3 (33.3%) (3, 25, 33)
DNA lesions—*in vivo* cytogenetics	4 (80.0%) (10, 14, 16, 32)	1 (20.0%) (25)

*In these comparisons, the drug gave only positive result (s) or only negative or inconclusive result (s) in the considered assays. In parentheses are indicated the number and corresponding percentages, as well as the numbers of Table 3*.

The highly consistent correlation between bacterial mutagenicity and gene mutation in mammalian cells indicated that the same genetic end point tests might have the high consistency. The discordance (nos. 32 and 45) may be due to the xenobiotic metabolism in the liver and other organs between the bacteria and animals. With the comparison between *in vitro* cytogenetics and *in vivo* cytogenetics, 2 (nos. 18 and 49) antiparasitics gave positive responses in *in vitro* cytogenetics while no. 13 gave negative. These results were inconsistent with that in *in vivo* cytogenetics. With regard to the discordant results between DNA lesions and *in vitro* cytogenetics of the three (nos. 3, 19 and 33) antiparasitics, two (nos. 19 and 33) antiparasitics tested negative and no. 3 yield positive in DNA lesions, respectively. These results were opposite to that in *in vitro* cytogenetics.

### A Novel Strategy for Predicting Carcinogenicity Based on the Genotoxicity Assays

Antiparasitics with both genotoxicity and carcinogenicity data are reported in Table 8 to analyze the correlation between the results of the various types of genotoxicity and carcinogenicity. The results are marked positive or negative or inconclusive. It is obvious that the concordant and discordant results occurred in all the 15 pairs of assays considered. When carcinogenicity in mice or rats was considered, the percentage of discordant results ranged from 71.4% between *in vivo* cytogenetics and carcinogenicity in both mice and rats to 10.0% between bacterial mutagenicity and carcinogenicity in both mice and rats. The rank order of the consistency between genotoxicity and carcinogenicity was bacterial mutagenicity > DNA lesions > *in vitro* cytogenetics > gene mutation in mammalian cells > *in vivo* cytogenetics.

**Table 8:** Correlation between the multiple genotoxicity and carcinogenicity in mice and rats assays of antiparasitics.

Couples of assays considered	No. of antiparasitics with	
	Concordant results	Discordant results
Bacterial mutagenicity—carcinogenicity in mice	11 (78.6%) (2, 3, 13, 15, 17, 19, 20, 25, 26, 33, 35)	3 (21.4%) (7, 10, 32)
Bacterial mutagenicity—carcinogenicity in rats	15 (93.75%) (2, 3, 7, 13, 15, 17, 20, 24–26, 30, 32, 33, 35, 45)	1 (6.25%) (10)
Bacterial mutagenicity—carcinogenicity in both mice and rats	9 (90.0%) (2, 3, 13, 15, 17, 20, 25, 33, 35)	1 (10.0%) (10)
Gene mutation in mammalian cells—carcinogenicity in mice	5 (55.6%) (19, 25, 26, 32, 33)	4 (44.4%) (7, 10, 36, 50)
Gene mutation in mammalian cells—carcinogenicity in rats	5 (50.0%) (7, 24, 25, 26, 33)	5 (50.0%) (10, 32, 36, 45, 50)
Gene mutation in mammalian cells—carcinogenicity in both mice and rats	3 (50.0%) (25, 26, 33)	3 (50.0%) (10, 36, 50)
*In vitro* cytogenetics—carcinogenicity in mice	7 (53.8%) (3, 13, 20, 24, 26, 32, 45)	6 (46.2%) (7, 15, 19, 25, 33, 50)
*In vitro* cytogenetics—carcinogenicity in rats	7 (58.3%) (3, 7, 13, 19, 20, 26, 30)	5 (41.7%) (15, 25, 32, 33, 50)
*In vitro* cytogenetics—carcinogenicity in both mice and rats	4 (50.0%) (3, 13, 20, 26)	4 (50.0%) (15, 25, 33, 50)
*In vivo* cytogenetics—carcinogenicity in mice	4 (36.4%) (26, 32, 35, 45)	7 (63.6%) (7, 13, 15, 17, 19, 25, 50)
*In vivo* cytogenetics—carcinogenicity in rats	5 (41.7%) (7, 19, 26, 30, 35)	7 (58.3%) (13, 15, 17, 24, 25, 32, 50)
*In vivo* cytogenetics—carcinogenicity in both mice and rats	2 (28.6%) (26, 35)	5 (71.4%) (13, 15, 17, 25, 50)
DNA lesions—carcinogenicity in mice	6 (75.0%) (20, 24, 25, 32, 33, 36)	2 (25.0%) (3, 30)
DNA lesions—carcinogenicity in rats	4 (57.1%) (20, 25, 33, 36)	3 (42.9%) (3, 32, 45)
DNA lesions—carcinogenicity in both mice and rats	4 (80.0%) (20, 25, 33, 36)	1 (20.0%) (3)

*In these comparisons, the antiparasitics gave only positive results or only negative or inconclusive results in genotoxicity assay and tested positive in at least one sex of mice or rats or gave negative or inconclusive results in both species in carcinogenicity assays. The following indicated the number and corresponding percentages, as well as the numbers of drugs of Table 3*.

Table 9 showed 2 types and 10 combinations of gene-tox assays based on bacterial mutagenicity to indicate the predictivity for rodent carcinogenicity. The sequence of the predictivity was (Ames–DNA lesions) = (Ames–DNA lesions–*in vitro*) = (Ames–DNA lesions–gene mutation in mammalian cells) = (Ames–*In vivo*–DNA) > (Ames–*in vitro*) = (Ames–*in vivo*) > (Ames–gene mutation in mammalian cells) > (Ames–*in vivo*–*in vitro*) > (Ames–gene mutation in mammalian cells–*in vivo*) = (Ames–gene mutation in mammalian cells–*in vitro*).

**Table 9:** Predictivity of multiple combinations with Ames for rodent carcinogenicity assays of antiparasitics.

Couples of assays considered	No. of antiparasitics with concordant results	Carcinogenicity		
		Concordant results	Discordant results	Without results
Ames–Gene	11 (6, 7, 10, 19, 20, 24–26, 28, 33, 42)	5 (62.5%) (19, 20, 25, 26, 33)	3 (37.5%) (7, 10, 24)	3 (6, 28, 42)
Ames–*In vitro*	16 (1, 3, 6, 7, 10, 13,16, 18, 20, 26, 28, 30, 36, 37, 42, 50)	6 (66.7%) (3, 13, 20, 26, 30, 36)	3 (33.3%) (7, 10, 50)	7 (1, 6, 16, 18, 28, 37, 42)
Ames–*In vivo*	13 (1, 6–9, 11, 16, 26, 30, 35, 36, 49, 50)	4 (66.7%) (26, 30, 35, 36)	2 (33.3%) (7, 50)	7 (1, 6, 8, 9, 11, 16, 49)
Ames–DNA	10 (16, 18, 20, 21, 25, 26, 30, 33, 36, 42)	6 (100.0%) (20, 25, 26, 30, 33, 36)	0	4 (16, 18, 21, 42)
Ames–Gene–*In vitro*	7 (6, 7, 10, 20, 26, 28, 42)	2 (50.0%) (20, 26)	2 (50.0%) (7, 10)	3 (6, 28, 42)
Ames–Gene–*In vivo*	3 (6, 7, 26)	1 (50.0%) (26)	1 (50.0%) (6)	1 (7)
Ames–Gene–DNA	5 (20, 25, 26, 33, 42)	4 (100.0%) (20, 25, 26, 33)	0	1 (42)
Ames–*In vitro*–*In vivo*	8 (1, 6, 7, 16, 26, 30, 36, 50)	3 (60.0%) (26, 30, 36)	2 (40.0%) (7, 50)	3 (1, 6, 16)
Ames–*In vitro*–DNA	7 (16, 18, 20, 26, 30, 36, 42)	4 (100.0%) (20, 26, 30, 36)	0	3 (16, 18, 42)
Ames–*In vivo*–DNA	1 (26)	1 (100.0%) (26)	0	0

*Ames, bacterial mutagenicity; Gene, gene mutation in mammalian cells; In vitro, in vitro cytogenetics; In vivo, in vivo cytogenetics; DNA, DNA lesions. In these comparisons, all the combinations took the Ames as center. The antiparasitics gave only positive results or only negative or inconclusive results in genotoxicity assay, and tested positive in at least one sex of mice or rats or gave negative or inconclusive results in both species in carcinogenicity assays. The following indicated the number and corresponding percentages, as well as the numbers of antiparasitics of Table 3*.

Table 10 presents the number and the percentage of antiparasitics that were classified as non-genotoxic non-carcinogens, genotoxic non-carcinogens, non-genotoxic carcinogens, and genotoxic carcinogens according to the genotoxicity assays considered. An antiparasitic was regarded as genotoxic when a positive response was given in at least one genotoxicity assay, and carcinogenic when it was tested positive in at least one rodent sex. Of the 20 antiparasitics with retrievable results of both genotoxicity and carcinogenicity, Malathion, Diazinon, Deltamethrin, Fenvalerate, Coumaphos, Tiabendazole, Albendazole, Cypermethrin, Amitraz and Praziquantel might be classified as genotoxic non-carcinogens; Fenthion, Lindane, Chlordimeform, Fipronil, Dichlorvos, Metronidazole, Pyrimethamine, and Imidacloprid can be classified as genotoxic carcinogens; Mefloquine was considered a non-genotoxic non-carcinogen, while the non-genotoxic carcinogens only contained Atovaquone, which tested negative in bacterial mutagenicity, *in vitro* and *in vivo* cytogenetic assays, but was found to induce liver tumors in mice in a long-term carcinogenesis assay (75, 122, 123).

**Table 10:** Correlation between the results of genotoxicity and carcinogenicity assays of antiparasitics.

Assay type	No. of non-genotoxic non-carcinogens	No. of genotoxic non-carcinogens	No. of non-genotoxic carcinogens	No. of genotoxic carcinogens
Ames	8 (42.1%) (2, 3, 13, 17, 19, 25, 33, 35)	1 (5.3%) (50)	6 (31.6%) (7, 10, 19, 24, 32, 45)	4 (21.1%) (20, 26, 30, 36)
Gene	2 (16.7%) (25, 33)	1 (8.3%) (50)	5 (41.7%) (7, 10, 19, 24, 36)	4 (33.3%) (20, 26, 32, 45)
*In vitro*	2 (11.1%) (3, 13)	6 (33.3%) (2, 15, 17, 25, 33, 50)	2 (11.1%) (7, 10)	8 (44.4%) (19, 20, 24, 26, 30, 32, 36, 45)
*In vivo*	1 (5.6%) (35)	7 (38.9%) (2, 13, 15, 17, 25, 33, 50)	2 (11.1%) (1, 20)	8 (44.4%) (10, 19, 24, 26, 30, 32, 36, 45)
DNA lesions	2 (15.4%) (25, 33)	2 (15.4%) (3, 15)	0	9 (69.2%) (10, 19, 20, 24, 26, 30, 32, 36, 45)

*Ames, bacterial mutagenicity; Gene, gene mutation in mammalian cells; *In vitro, in vitro* cytogenetics; *In vivo, in vivo* cytogenetics. The data show the number of antiparasitics that classified as non-carcinogens and carcinogens, which were examined in each genotoxicity assay and the result was negative (non-genotoxic) and positive (genotoxic) in the same assay. In this analysis, the antiparasitics that did not increase tumor incidence in mice and/or rats of both sexes were considered as non-carcinogens, and that increased tumor incidence in at least one sex of mice or rats were considered as carcinogens. An antiparasitic was considered non-genotoxic when it gave a single negative result, and genotoxic when it gave a single positive or concordant positive result in the indicated genotoxicity assay. The following indicated the number and corresponding percentages, as well as the numbers of antiparasitics of Table 3*.

The bacterial mutagenicity has the highest specificity but the lowest sensitivity (Table 8), while DNA lesions (*in vitro* and/or *in vivo*) have the highest sensitivity and a lower specificity. A test with a low specificity induced a high proportion of misleading positive results. Therefore, the combination of bacterial mutagenicity and DNA lesions has high accuracy in relation to rodent cancer, which is consistent with the above analysis results. A proportion of 5.3% of antiparasitics gave positive in bacterial mutagenicity and was classified as non-carcinogens. There were 31.6% of antiparasitics that were regarded as carcinogenic while gave a negative result in bacterial mutagenicity.

## Discussion

The economic importance of parasitic infections in livestock and humans has long been recognized. Meanwhile, the most important advances in antiparasitics have come from the animal health area. Although many antiparasitics have been developed and applied to control parasitism in humans and animals, genotoxicity and carcinogenicity studies have not been conducted on a large proportion of them. Since a relationship between exposure to genotoxic compounds and carcinogenesis has been established, genotoxicity tests have been proposed for all medicinal products for human use except for some compounds (e.g., anticancer) that can interact with DNA (11). Therefore, this review was to assess the extent of antiparasitics that have been tested for genotoxic and carcinogenic activity. In addition, the ability of various types of genotoxicity assays was summarized to discriminate rodent carcinogens, which benefit to analyze the relative predictivity of carcinogenicity in rodents and humans. Furthermore, it is necessary to re-evaluate *in vitro* genotoxicity according the present revised guidelines.

With regard to the genotoxicity assays, compared to the positive and discordant results, the incidence of negative responses is 61.7, 61.1, 21.2, 25.8, and 15.8% for bacterial mutagenicity, gene mutation in cultured mammalian cells, *in vitro* cytogenetics, *in vivo* cytogenetics, and DNA lesions (*in vitro* and *in vivo*), respectively. It was observed that the incidence of negative responses was higher than the positive and discordant results in bacterial mutagenicity and gene mutation in cultured mammalian cells. Kasper et al. (240) reviewed the advantages and limitations of the standard genotoxicity tests in predicting the ability and the mode of action for carcinogens, which demonstrated that a totally negative response in all the standard genotoxicity assays was sufficient to prove the non-genetic toxicity of the chemicals, while the presence of a positive response in some genotoxicity assays, particularly in Ames and *in vitro* genotoxicity studies, did not afford support for the genetic definition of the chemicals. There have been a number of experiences in the literature regarding the high correlation among the various types of genotoxicity assays with respect to carcinogens (241, 242), which suggested that a chemical that tested positive in *Salmonella* tended to yield positive responses in any other *in vitro* genotoxicity studies, for instance, chromosome aberrations (CA), SCEs, and mutations in mouse lymphoma cells (MLA) (243).

A high percentage of antiparasitics tested positive in the following assays: *in vitro* cytogenetics, *in vivo* cytogenetics, and DNA lesions (*in vitro* and *in vivo*). It is worth noting that the proportion of positive responses in *in vitro* cytogenetics is higher than in other types of assays. The *in vitro* cytogenetics seems to be more sensitive to genetic substance. However, the *in vitro* assays always lead to a number of false-positive results in genotoxicity and the carcinogenicity in rodents (244, 245). It was learned from the literature that the massive positive results only occurred at high levels of concentration. Recent surveys for *in vitro* cytogenetics were taken from compilations such as that of Müller et al. (246), Kirkland and Müller (247), Müller and Kasper (248), and Hilliard et al. (249). The conclusion was that the highest testing concentrations might lead to an increase in the emergence of misleading, toxicity-related positive results. In cytotoxicity and chromosome aberrations *in vitro*, Galloway (250) found that the positive response in genetic toxicology was caused by the cytotoxicity rather than the true drug or DNA interactions. Parry et al. (251) examined 24 carcinogens that gave positive results in *in vitro* genotoxicity at 1–10 mM, yet almost half of them were not mechanistically genotoxic carcinogens or had carcinogenic effects only in excessive doses. In the present review, we re-evaluate the *in vitro* genotoxicty according to current ICH S2 (R1) guidance. We find that the percentage of antiparasitics in agreement with the current ICH S2 (R1) guidance for *in vitro* genotoxicity data acceptance was 15 (45.5%). Thus, it is essential to re-evaluate *in vitro* genotoxicty that conducted prior to the update guideline of ICH S2 (R1) to provide a comprehensive assessment of the genotoxic effects.

Misleading positive results were found not only in *in vitro* but also in *in vivo* genotoxic assays. Increasing experience suggested that the occurrence of a positive response in rats and mice micronucleus tests was not the consequence of intrinsic genotoxicity but drug-related disturbances in the physiology (252), such as lysosomal damage, ATP depletion or impairment of mitochondrial function and the release of DNA endonucleases. However, at the time of writing, there has still been no amendment to the guidelines requirements of *in vivo* genotoxicity for dose limitations and toxicity to avoid irrelevant physiological responses. Furthermore, there is no consensus as to the highest testing concentration in *in vitro* genotoxicity assays. The method for the detection of toxicity has greatly changed in recent years, and the limitations of dose and toxicity in genotoxicity testing in OECD and ICH should be adjusted to adapt to the new changes. The standard genotoxicity system also needs to identify the cytotoxicity and genotoxicity clearly.

There are many explanations that could account for the existence of different results in the various types of genetic tests. The differences are the following: the detection of the genetic end point; the xenobiotic metabolism between bacterial mutagenicity and mammalian cells; the effective dose between *in vitro* and *in vivo*, especially the *in vivo* decomposition; the relative sensitivities of various genotoxicity assays to genetic damage; the metabolic activation pathway and metabolizing enzymes among species. *In vivo* activity, which is designed to study the mechanisms of mutagenicity in the potential target organs of rodents, is the best method to confirm the differences in cytogenetics between *in vivo* and *in vitro*. Except for the irrelevant biological reaction at high doses, it is also accepted that the metabolic activation process and metabolites could induce genetic toxicity. Some evidence suggested that the genetic toxicity of compounds may be prototypes or metabolites. For the drugs that are theoretically nitrosatable in the presence of amine, the interaction resulted in the formation of genotoxic–carcinogenic N-nitroso compounds (253). However, the current standard of genotoxicity assays cannot distinguish whether the positive results are derived from the drugs or their metabolites directly.

In Table 7, the percentage of concordant results between bacterial mutagenicity and carcinogenicity in both mice and rats is 90.0%, which is higher than any other correlation pairs. The same conclusion was drawn by Snyder and Green (19) in a review of the genotoxicity of marketed pharmaceuticals. Data from 467 marketed drugs were collected and no combination of gene-tox assays provided a higher predictivity of rodent carcinogenesis than the bacterial mutagenicity test itself (19). In two studies conducted by Zeiger, one identified 172 chemicals that gave negative or equivocal results in 2-year rodent assays, yet 38 (22.1%) chemicals produced positive results in *Salmonella* (243). Another found that among 158 drugs that tested negative in carcinogenicity assays, 33 (21%) were *Salmonella* mutagens (254). However, a chemical that tested negative in *Salmonella* testing cannot be regarded as a non-carcinogenicity because the percentage of rodent carcinogens that are not mutagenic is about 50% (254). It was also reported that the predictivity for rodent carcinogenicity of bacterial mutagenicity ranged from approximately 77 to 98% (254, 255). The remaining 2–23% was classified as non-carcinogen with positive result in bacterial mutagenicity, which demonstrated the flaw and insufficiency on the prediction carcinogenicity of bacterial mutagenicity.

Therefore, it requires efforts to overcome the deficiencies of bacterial mutagenicity and improve the predictivity for carcinogenicity. We try to find which genotoxicity assay(s) considered could enhance the prediction of bacterial mutagenicity to rodent carcinogenicity. Our approach has many differences and improvement compared to Snyder and Green (19), who examined only five combinations of gene-tox assays, such as Ames–*in vitro* cytogenetics, Ames–*in vivo* cytogenetics, *In vitro* cytogenetics–*in vivo* cytogenetics, MLA–*in vivo* cytogenetics, and MLA–*in vitro* cytogenetics (19). These combinations have no DNA lesions tests and no taking bacterial mutagenicity as center. A review suggested that DNA lesion alone could contribute to the prediction of carcinogenicity in mice (255). In the present article, as shown in Table 8, DNA lesion testing can significantly increase the predictivity of Ames from 90 to 100%, suggesting that the combination of DNA lesions and bacterial mutagenicity obtained higher prediction of carcinogenicity.

There are three types of DNA lesions: (a) the formation of DNA adducts; (b) DNA repair synthesis (UDS); and (c) the induction of DNA strand breaks and cross-links. An analysis of correlations between the induction of DNA lesions and carcinogenic activity was conducted in 2010 (256). It noted that the carcinogenic activity of some drugs can be correctly predicted by DNA lesion assays, yet neglected in the standard 3-test battery. Thus, DNA lesion assays were considered the best supplement for the standard 3-test battery. The occurrence of the highest predictivity in a combination of bacterial mutagenicity and DNA lesions in our review suggested a close relationship between genotoxicity and carcinogenic activity. The bacterial mutagenicity test was often used to measure the ability of a drug to cause mutations rather than a definitive test of the carcinogens. The *in vivo* DNA lesion tests can detect the chemicals that reach the appropriate target with an effective dose to convert into a permanent mutation by reacting with DNA. In a few cases, the mutation escaped monitoring to survive and subsequently, carcinogenicity was generated through a loss of restriction of cell division. The *in vivo* DNA lesions can identify this “survived mutation.” Thus, the combination of bacterial mutagenicity and DNA lesions showed a higher and more accurate predictivity of carcinogenicity.

The correlation between the results of genotoxicity and carcinogenicity assays of antiparasitics was indicated in Table 9. Among the antiparasitics that were classified as genotoxic carcinogens, 69.2% tested positive in *in vitro* and/or *in vivo* DNA lesions exhibiting a greater sensitivity to carcinogens than any other types of genotoxicity assays. Eight out of 19 (42.1%) antiparasitics gave negative results in bacterial mutagenicity and were identified as non-carcinogens. Sensitivity and specificity are commonly used to describe the capability of *in vitro* genotoxicity assays (257). Sensitivity is defined as the percentage of genotoxic carcinogens that produced positive results in the considered test, and specificity is regarded as the ratio of non-carcinogens that gave negative responses. The ability of a battery of three *in vitro* genotoxicity tests to discriminate between rodent carcinogens and non-carcinogens was made by Kirkland et al. to increase the specificity of a valid test (258). The conclusion was that the “profile” of the genotoxicity results, such as the concentration, the level of toxicity and magnitude of response, provided a body of evidence to predict the carcinogenic results (259).

The rodent bioassays were useful and relevant for predicting risks of human cancers (260). The epigenetic changes with a loss of restriction of cell division (261) and the DNA oxidative stress damage were likely to produce cancer. Trosko and Upham found that the changes in gene expression caused by cell communication systems play a key role in the imbalance of cell proliferation, differentiation, and apoptosis, eventually promoting the tumor process (262). A large number of rodent tumor findings were found not relevant for humans (262) recently. It is worth noting that traditional carcinogenicity studies are largely not predictive of human cancer risk, therefore the well-suited approaches were proposed, for instance, the genetically modified animal models (15), and *in vitro* carcinogenicity screening assays based on gene expression profiling (16, 263). From the perspective of prospects, a more useful and accurate method to predict the carcinogenicity in humans is very urgent.

Herein, 136 antiparasitics were collected from both human and veterinary pharmacopeia. Due to the design of toxicity and the highest concentration in *in vitro* genetic toxicity tests have changed enormously in current guidelines, the reliably of old data were evaluated and as low as 45.5%. For a larger proportion of antiparasitics, whose genotoxicity and/or carcinogenicity results were not retrievable, the retesting based on revised guidelines should be done to make a safety assessment of human health. The combination of DNA lesions and bacterial mutagenicity is more accurate for predicting carcinogenicity than bacterial mutagenicity alone or together with any other genotoxicity testing. Development of this method for predicting carcinogens should be applied to reduce the misleading hazard alerts of the new and effective drugs.

## Author Contributions

ZY conceived the idea. XW analyzed and discussed data. QL analyzed and discussed data and wrote the article. ZL performed and revised the experiments. AI and FZ revised the article. All the authors discussed the results and contributed to the final manuscript.

## Conflict of Interest Statement

The authors declare that the research was conducted in the absence of any commercial or financial relationships that could be construed as a potential conflict of interest.
